# Fire at the boundary: Airborne toxic metals from the 2025 Los Angeles urban firestorm

**DOI:** 10.1126/sciadv.aei4455

**Published:** 2026-10-28

**Authors:** Haroula D. Baliaka, Ryan X. Ward, Sean M. Raffuse, Bradley Ries, Yumeng Cui, Dien Wu, Ruizhe Liu, Can Barut, Sina Hasheminassab, François L. H. Tissot, Armistead G. Russell, John H. Seinfeld, Richard C. Flagan, Roya Bahreini, Paul O. Wennberg, Nga L. Ng

**Affiliations:** ^1^ California Institute of Technology, Pasadena, CA 91125, USA.; ^2^ University of California, Davis, Davis, CA 95616, USA.; ^3^ University of California, Riverside, Riverside, CA 92521, USA.; ^4^ Colorado State University, Fort Collins, CO 80523, USA.; ^5^ Georgia Institute of Technology, Atlanta, GA 30332, USA.; ^6^ Jet Propulsion Laboratory, California Institute of Technology, Pasadena, CA 91109, USA.

## Abstract

Urban fire smoke is chemically distinct from wildland fire smoke yet remains poorly understood. Using real-time airborne trace element measurements during the 2025 Los Angeles Eaton urban firestorm, we show that the plume evolved rapidly as fire transitioned from structures to wildland, tracked hourly by the potassium-to-lead ratio. Compared to wildfire smoke, the urban plume was enriched more than 50-fold in lead, 7-fold in halogens, and 3-fold in arsenic, a toxic mixture linked to burning vehicles, electronics, and building materials, a fundamentally different fuel from conventional wildland vegetation. During peak structural burning, we estimate that approximately 7 million residents (range, 4.3 to 8.0 million) experienced 24-hour lead concentrations exceeding 50 nanograms per cubic meter, over 80 times background levels. Unlike wildfire smoke that has been the focus of decades of air quality research, this plume represents an emerging class of urban fire air pollution that demands revised exposure frameworks and emergency response protocols as fires increasingly burn into cities.

## INTRODUCTION

For decades, the word “wildfire” has evoked images of forests burning, but this perception is shifting with the growth of the wildland-urban interface (WUI) (*1*), the transition zone where human development meets wildland vegetation. The Camp Fire (2018) (*2*, *3*), Marshall Fire (2021) (*4*), Lahaina Fire (2023) (*5*), and now the Eaton and Palisades fires (2025) (*6*, *7*) are examples of how the traditional picture of forests burning has markedly changed. On 7 January 2025, the Eaton Canyon ignited in the foothills above Altadena, one of the oldest residential communities in Los Angeles. Driven by Santa Ana winds with gusts exceeding 100 mph (*8*, *9*), the fire escalated within a few hours into an urban firestorm that destroyed more than 9000 structures. This recent fire was the second most destructive fire in California history (*10*). The Los Angeles basin was covered in dense smoke plumes for several days, exposing millions of residents to not only elevated fine particulate matter (PM_2.5_) (*11*) but also a cocktail of chemicals (*12*). Unlike the wildfire smoke that has been the focus of decades of air quality research (*13*–*16*), this plume originated primarily from burning homes, vehicles, and infrastructure, a fundamentally different fuel with a distinct chemical signature (*4*, *12*, *17*–*19*).

Wildfire PM_2.5_ derives largely from vegetative combustion and has been linked to respiratory and cardiovascular effects, contributing to an estimated 1.5 million premature deaths annually from landscape-fire smoke worldwide (*13*–*16*). But when structures burn, they become sources of airborne particle–bound metals, halogenated compounds, and other species associated with synthetic building materials, electronics, and treated wood, many of which are toxic even at trace concentrations (*19*–*21*). This chemical mixture is particularly concerning in Los Angeles, where much of Altadena’s housing inventory dates back to the early-to-mid 20th century (*22*), when leaded paint or brominated flame retardants were still common.

The chemical signature that distinguishes urban fire plumes from wildfire smoke and the health risks it may pose remain poorly understood and have been identified as a critical research need by the National Academies of Sciences, Engineering, and Medicine (*17*). This gap persists in part because, while regulatory monitoring networks measure total PM_2.5_ mass at high temporal resolution, they rely on time-integrated, filter-based sampling for chemical speciation. These samples are typically collected over 24 hours, often every third or sixth day, and are analyzed weeks to months after collection, limiting their ability to capture rapid changes in chemical composition during episodic events such as fires or sometimes missing them entirely. As a result, while total PM_2.5_ concentrations can be tracked in near real time, information on chemically resolved components, including toxic species such as lead (Pb) and chromium (Cr), is often not available at high-time resolution during active events. In parallel, emission models have primarily relied on biomass combustion assumptions that fail to capture the chemical complexity of urban fire emissions. Consequently, emission inventories likely underestimate toxic exposures, and communities remain unaware of immediate chemical risks during WUI fire events (*17*).

Here, we present high temporal resolution PM_2.5_ trace element measurements of the Eaton fire plume reported in real-time by the Atmospheric Science and Chemistry mEasurement NeTwork (ASCENT), a recently established network providing continuous, in situ chemical speciation measurements of ambient PM_2.5_ (*23*). These observations enable us to resolve rapid changes in smoke composition during a dynamic fire event: We (i) characterize the elemental composition of the fire plume, (ii) track how this composition evolved over the course of the fire, and (iii) quantify source impacts to distinguish wildland fire–derived PM_2.5_ from emissions associated with the burning of structures and background sources. From this analysis, we estimate population exposure to trace metals, particularly focusing on Pb, across the Los Angeles Basin during the first few days of the fire. These results provide new insights for emission inventories and inform health risk exposure assessments for similar WUI fire events in the future.

## RESULTS

### Urban fire plumes exhibit distinct chemical signatures compared to wildfire smoke

Continuous elemental measurements of PM_2.5_ were collected at the ASCENT monitoring site in Pico Rivera, Los Angeles. Details of the site and analytical methods are provided in the Supplementary Materials. To establish how the elemental signature of urban fire smoke differs from that of a wildland fire plume, we compare the Eaton fire to a wildland fire that affected a nearby ASCENT site a few months earlier. Specifically, we use data from the Rubidoux site in Riverside, CA during a fire in September 2024 as an illustrative example of a predominantly wildland plume and contrast it to the Eaton fire smoke measured at the Pico Rivera site in January 2025 (Fig. 1, A and B). For both events, we quantify fire enhancement ratios relative to background levels measured several days before each event for the suite of trace elements meeting our detection criterion, as well as total PM_2.5_ mass (Fig. 1C; see sections S2.1 and S3 and tables S1 and S2).

**Fig. 1.**
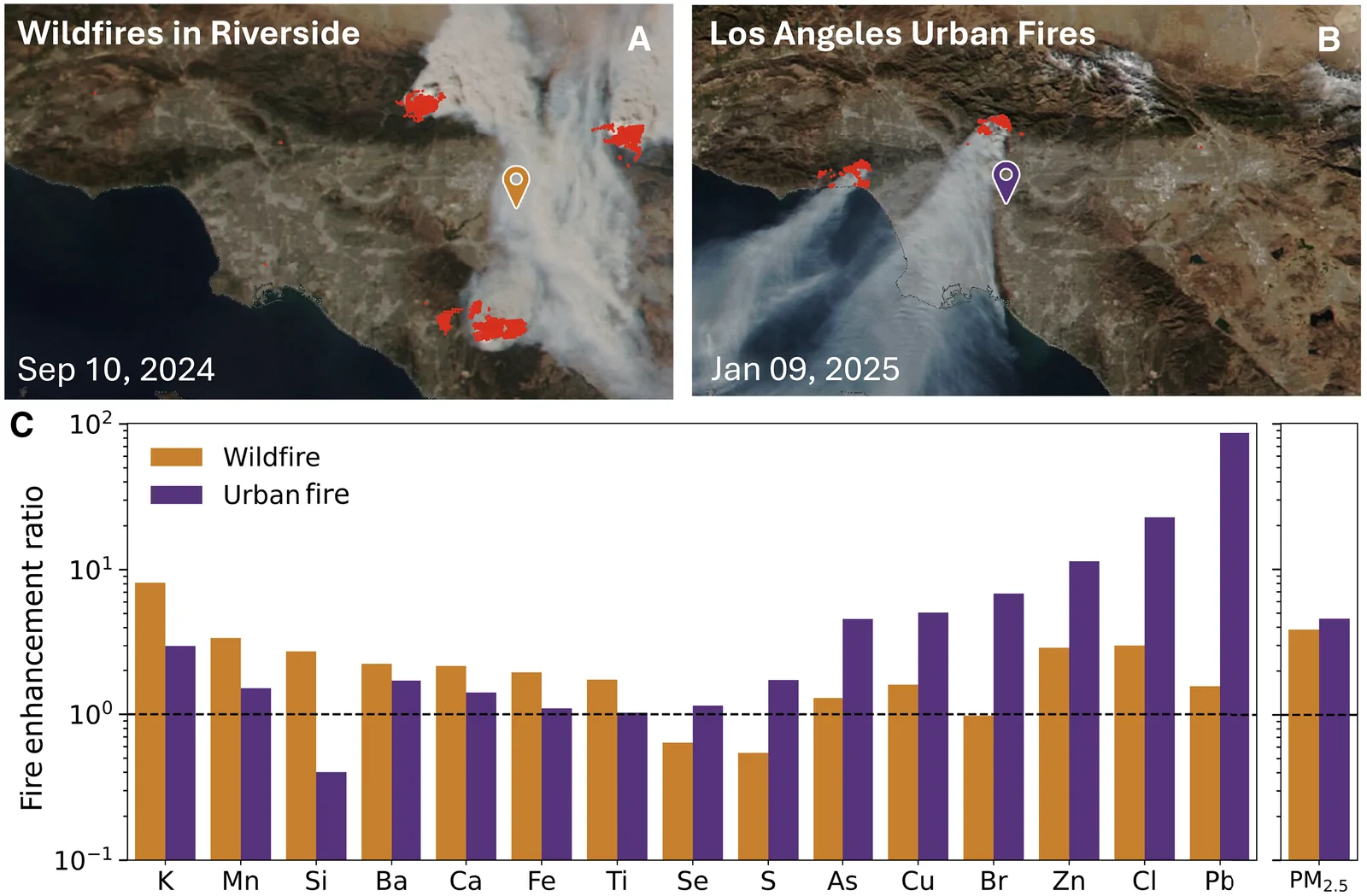
Urban fire plumes exhibit distinct chemical signatures compared to wildfire smoke. (**A**) NOAA-20 satellite image of the wildfire plume observed on 10 September 2024. The orange location pin denotes the Rubidoux monitoring site in Riverside, CA. (**B**) NOAA-20 satellite image of the urban fire plume over Los Angeles on 9 January 2025, with the Pico Rivera site marked by a purple pin. Red markers denote VIIRS active fire/thermal anomalies. (**C**) Fire enhancement ratios (plume period over background period) for PM_2.5_ concentrations and the retained suite of measured elements (ordered by decreasing wildfire enhancement ratio (K through Ti) and increasing urban fire enhancement ratio (Se through Pb) comparing the wildfire (based on the Rubidoux measurements) and urban fire (based on the Pico Rivera measurements) plumes.

PM_2.5_ mass enhancements were similar between the two fires (*24*, *25*); their chemical compositions, however, tell a different story. As expected, both wildland and urban fire plumes show elevated levels of potassium (K), a tracer for wood burning that was first described in the 1980s (*26*, *27*). However, the urban fire plume is enriched in several additional elements commonly associated with anthropogenic sources (*28*). Chlorine (Cl) and bromine (Br) were more than sevenfold higher relative to the wildfire, while zinc (Zn) showed fourfold enrichment and copper (Cu) and arsenic (As) more than threefold. Most notably, Pb concentrations were more than 50-fold higher during the urban fire relative to the wildfire signature.

### The dynamic nature of the Eaton urban fire captured by real-time measurements of airborne trace elements

To further explore the chemical contrast between urban and wildland fire plumes, we take the two tracer elements at opposite ends of Fig. 1C, K and Pb, and examine their relationship as a potential indicator of structure-dominated versus biomass-dominated combustion. Figure 2B shows hourly K and Pb concentrations at the Pico Rivera site from 8 to 13 January 2025. Both tracers peak simultaneously on 9 January, coinciding with the most intense period of plume transport from the Eaton Canyon fire. In Fig. 2C, we plot K against Pb for both the Pico Rivera urban fire plume and the Rubidoux wildfire plume, colored by measurement date. The wildfire plume clusters at high K/Pb ratios (≈50–500), consistent with potassium-dominated biomass combustion. In contrast, the early urban fire plume exhibits ratios as low as K/Pb ≈ 5, reflecting enhanced Pb release from the combustion of anthropogenic materials.

**Fig. 2.**
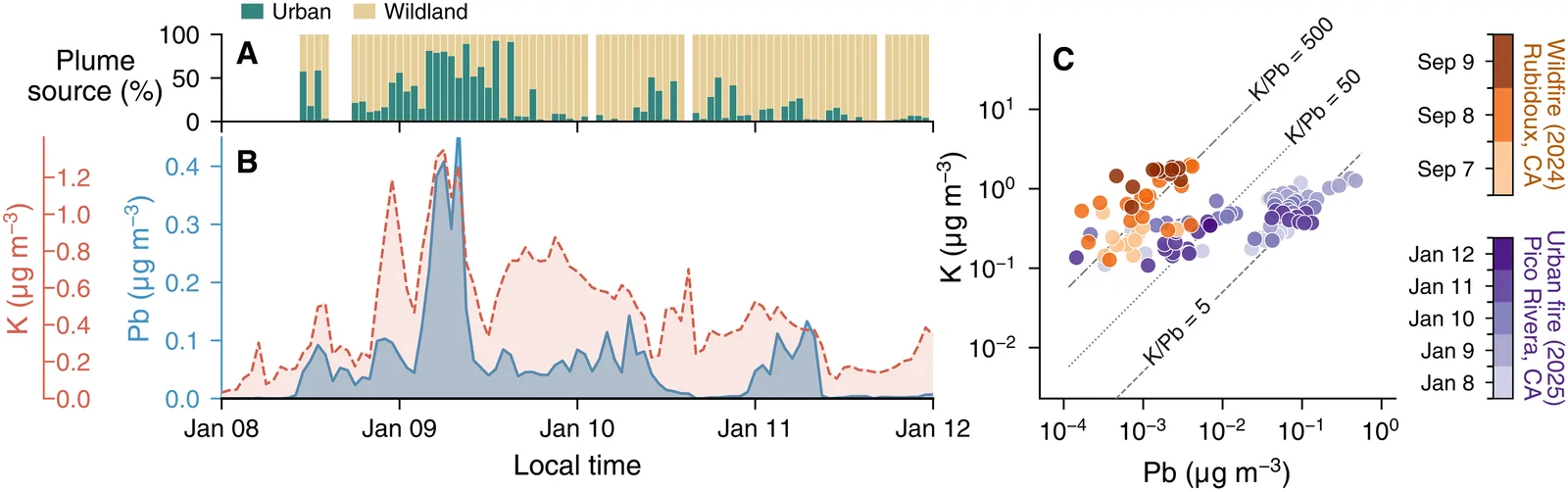
Potassium (K) to lead (Pb) ratios track the transition from urban to wildland fire emissions. (**A**) Plume source origin (percent) at the Pico Rivera receptor site, estimated using the STILT atmospheric dispersion model, shows the relative contributions of urban and wildland burning. (**B**) Hourly time series of K (dashed line) and Pb (solid line) at Pico Rivera during the January 2025 Eaton fire. (**C**) K versus Pb scatterplot comparing the Pico Rivera urban fire plume (purple) with the 2024 wildfire plume measured at Rubidoux (orange). Gray dashed lines denote constant K/Pb mass ratios of 500, 50, and 5. The early urban fire plume shows distinctly lower K/Pb (∼5) due to enhanced Pb release from anthropogenic materials. As the fire progresses and biomass burning becomes dominant, the K/Pb ratio shifts upward toward wildfire-like values, consistent with the transition to wildland-dominated plume source shown in (A).

What is perhaps most telling is the temporal evolution within the Eaton fire itself. The fire’s chemistry shifted rapidly as its path burned through neighborhoods in the first 48 hours and then transitioned north into the open wildland of the San Gabriel Mountains as winds subsided, with the composition of the smoke changing hour by hour (*9*). This transition is evident in the real-time K/Pb ratio at Pico Rivera, which steadily shifted upward over the following days, converging toward wildfire-like values. This chemical transition is further corroborated (Fig. 2A) by the Stochastic Time-Inverted Lagrangian Transport (STILT) atmospheric dispersion model (see section S5), which shows that the urban plume source fraction at Pico Rivera declined sharply after the first 2 days.

### Source apportionment of the urban fire plume: Separating background and resolving fire-enhanced sources

Urban fire plumes comprise a mixture of chemically distinct aerosol classes associated with different combustion sources. We applied source apportionment analysis to the full suite of PM_2.5_ elements and identified a five-source solution (Fig. 3) to be the most statistically robust and physically interpretable (see section S6).

**Fig. 3.**
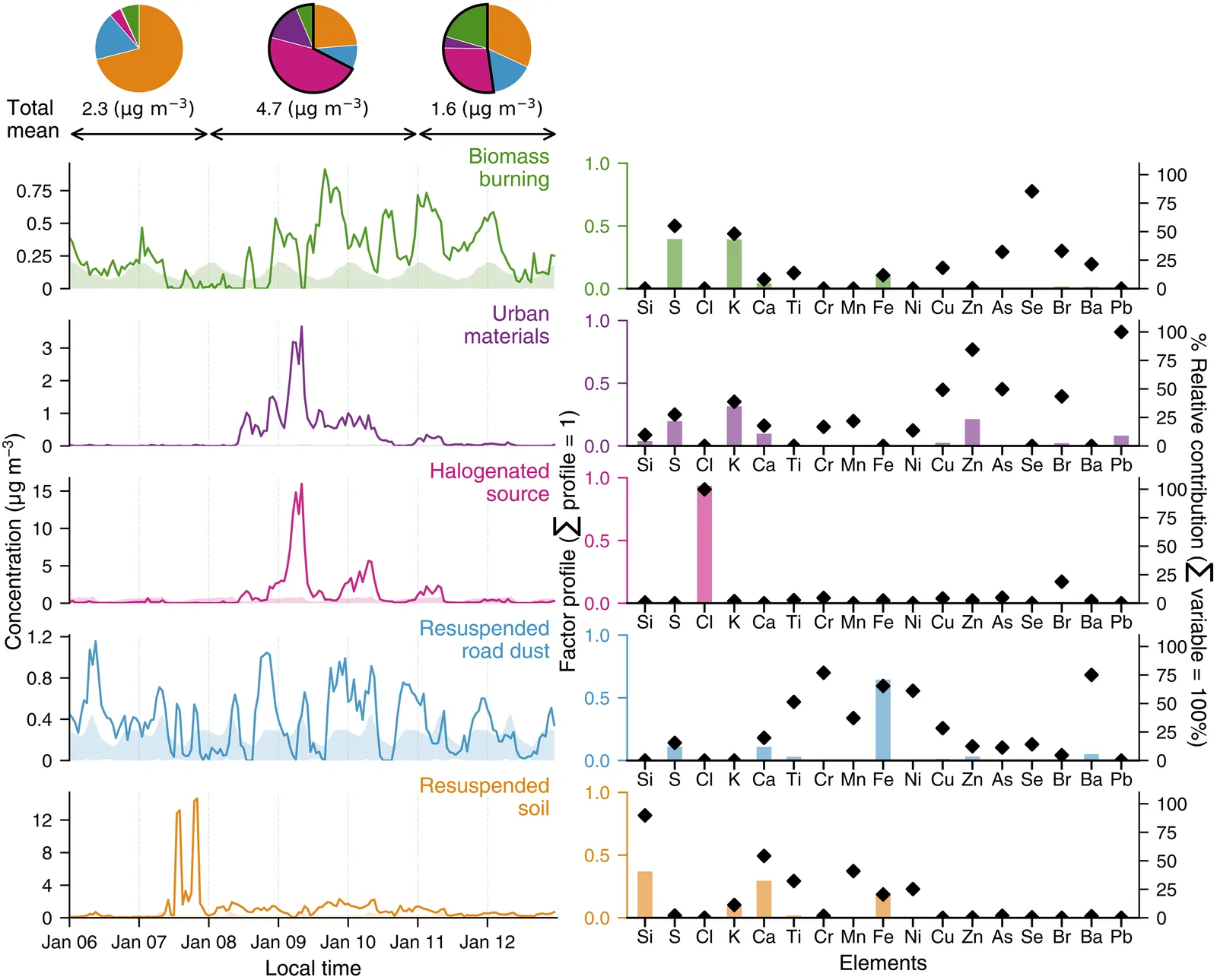
Source apportionment of PM_2.5_ elements during the fire-impacted week (6 to 12 January 2025). **Left**: variation in concentrations for five sources: biomass burning, urban materials, halogenated source, resuspended soil, and resuspended road dust. Solid lines show the time series of each source during the fire period. Shaded areas (±1σ) correspond to the mean background concentrations from September 2023 to December 2024 (see the Supplementary Materials). The pie charts demonstrate mean source contributions for three distinct periods with total mean concentrations indicated below each chart (2.3, 4.7, and 1.6 μg m^−3^, respectively); wedges outlined in black denote fire-enhanced sources. **Right**: factor profiles. For each factor, bars (left axis, ∑ = 1 within panel) show how that factor’s mass is distributed across elements: i.e., the factor’s chemical fingerprint. Black diamond markers (right axis, ∑ = 100% across factors for a given element) show how much of each element’s total mass is attributable to that factor.

Two background sources are resolved; they are both present before and throughout the fire period. Resuspended road dust, enriched in barium (Ba) and iron (Fe), shows no temporal correlation with fire intensity and follows the typical daily traffic pattern. Resuspended soil, enriched in silicon (Si) and calcium (Ca), spikes briefly on 7 January due to wind-driven dust during the Santa Ana event but otherwise remains decoupled from fire activity.

There are three “fire-enhanced” sources, each exhibiting distinct chemical signatures and temporal behavior. Biomass burning, which is dominated by K and sulfur (S), is elevated throughout the fire period and persists as the fire moves into wildland terrain. The halogenated source is dominated by Cl and accounts for the largest share of fire-enhanced elemental PM_2.5_ during the peak urban plume. Cl-containing materials such as polyvinyl chloride are ubiquitous in the built environment and have been identified as a priority knowledge gap for WUI fire emissions (*17*, *19*). Last, an urban materials source shows a sharply different temporal profile: It peaks on 8 and 9 January, coinciding with the most intense period of structural burning, and then declines rapidly. This factor accounts for 100% of the measured Pb and contains elevated As, Br, Cu, and Zn; these elements are associated with the combustion of vehicles, electronics, and a broad range of human-made products, including building materials and synthetic furnishings (*17*, *19*). Together, these results define structure fire emissions as distinct aerosol classes with unique elemental fingerprints.

### Millions of residents exposed to Pb-containing PM_2.5_ from urban materials burning

The Pb time series is perhaps the most notable of the elements in the urban materials source: Hourly concentrations peaked at 0.47 μg m^−3^, more than three times the EPA National Ambient Air Quality Standard of 0.15 μg m^−3^ (3-month rolling average) (see fig. S6). Because Pb was concentrated in the urban materials factor and it is a highly toxic air contaminant (*29*) with substantial health risks (*30*, *31*), particularly neurodevelopmental effects in children (*32*), we use it here as a practical tracer of potential population exposure to this broader toxic mixture. To obtain a first-order estimate of basin-wide Pb exposure, we integrated colocated PM_2.5_ measurements (from Pico Rivera, Rubidoux, and Joshua Tree) into a spatial fusion model combining real-time observations with a smoke forecast model to map daily Pb concentrations at 1-km resolution across the Los Angeles basin (see section S7) (*33*).

As shown in Fig. 4A, the highest 24-hour average Pb concentrations (>70 ng m^−3^) occurred across eastern Los Angeles and the San Gabriel Valley on 8 and 9 January, with peak values in communities directly downwind of Altadena. Under typical conditions, daily Pb at Pico Rivera averages just 0.6 ng m^−3^ (see fig. S6). Overlaying daily Pb maps with census data reveals the scale of exposure: On 9 January, approximately 7 million residents (range, 4.3 to 8.0 million) experienced concentrations exceeding 50 ng m^−3^ (∼80× typical) (Fig. 4B). Notably, as peak concentrations declined with the transition from structural to wildland burning, total population exposed continued to rise as the plume dispersed across the basin.

**Fig. 4.**
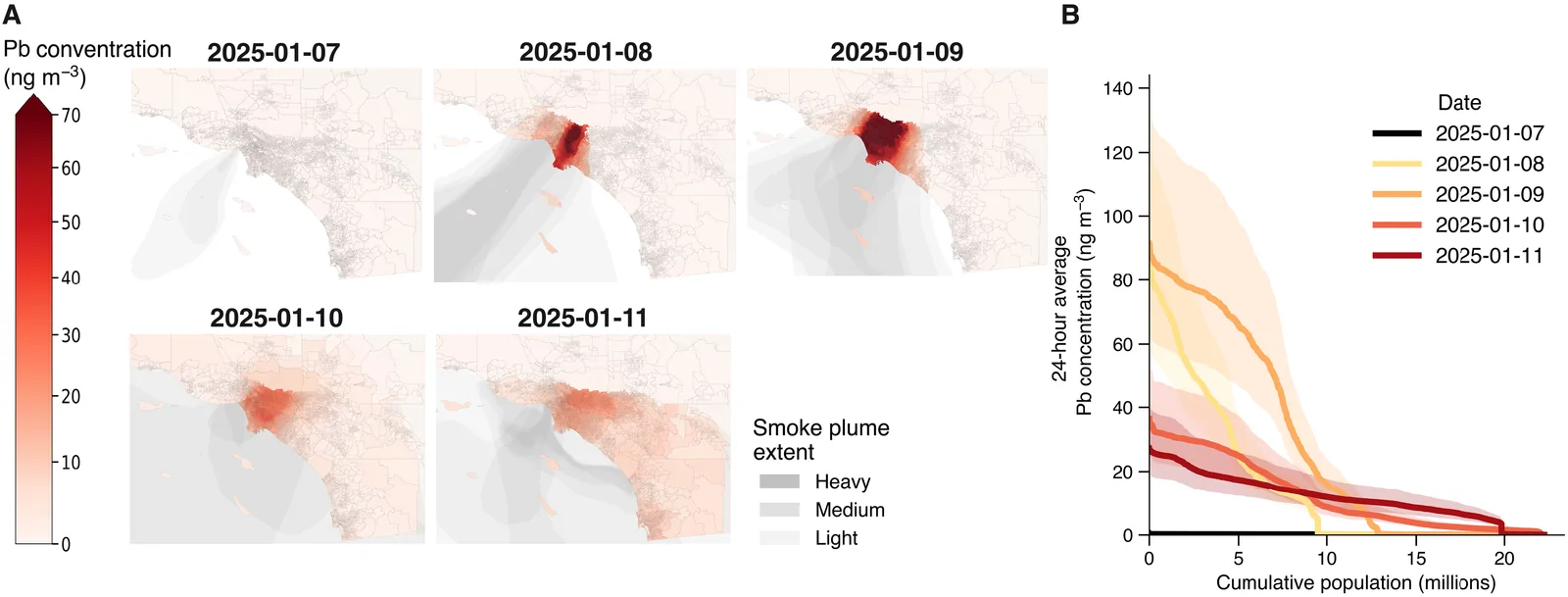
Spatiotemporal evolution of estimated atmospheric Pb exposure during the January 2025 Los Angeles fires. (**A**) Daily 24-hour average Pb concentrations mapped at 1-km spatial resolution over Los Angeles and surrounding regions from 7 to 11 January 2025. Darker colors indicate higher concentrations. (**B**) Cumulative population exposed to 24-hour average Pb concentrations for each day. Shaded bands denote the model bias range (0.71 to 1.49×), defined as the minimum and maximum of the observed-to-modeled Pb ratio at the Pico Rivera validation site during the fire period (8 to 11 January 2025) (see section S7.4).

To place these values in historical context, ambient Pb concentrations in the Los Angeles basin have declined by more than two orders of magnitude since the phase-out of leaded gasoline, from routine values of more than 1 μg m^−3^ in total suspended particles in the early 1980s to background levels of roughly 5 to 20 ng m^−3^ over the past two decades (see fig. S7). For Pico Rivera, the highest 24-hour average Pb concentration of 0.12 μg m^−3^ and the hourly peak of 0.47 μg m^−3^ measured during the Eaton fire therefore represent a marked temporary departure from the modern baseline, delivering a short-lived but concentrated exposure whose health consequences remain poorly characterized.

## DISCUSSION

Urban firestorms represent a chemically distinct class of air pollution for which existing air quality monitoring infrastructure was not designed to provide real-time, chemically resolved measurements, and current public health systems have limited information for assessing these rapidly evolving exposures. The Los Angeles Eaton urban fire, with its unprecedented combination of scale and population exposure, illustrates how a single firestorm can transform an entire metropolitan airshed within hours. Our results track the real-time chemical evolution of an urban fire plume. We show how this chemical signature evolved rapidly as the fire transitioned from structures to wildland burning in the days that followed.

What makes urban fire smoke distinct is not just its chemistry, but its potential toxicity. The health impacts of the metal- and halogen-rich mixtures unique to fires in the built environment are a substantial and largely unaddressed gap (*17*, *19*). While the toxicity of chronic exposure to individual elements [e.g., neurotoxicity of Pb (*30*, *32*) or carcinogenicity of As (*34*)] is well established, far less is known about the consequences of these short-duration but high-intensity exposures, as well as the cumulative effects of simultaneous exposure to complex mixtures. The urban materials source we identified contains multiple elements of toxicological concern, including EPA-designated hazardous air pollutants (e.g., Pb and As) (*35*). There is an urgent call for epidemiological studies to explore the health outcomes associated with these exposures, particularly in children and other vulnerable populations.

Impacts of urban fire emissions extend beyond inhalation during active burning (*36*, *37*). Once released, this toxic mixture is transported over hundreds of miles, depositing contaminants across playgrounds, watersheds, reservoirs, and agricultural land. The large copresence of halogens in the already polluted Los Angeles basin (*38*–*40*) creates conditions favorable for atmospheric oxidative chemistry that could alter aerosol composition and toxicity well beyond the immediate fire zone (*41*–*43*). Ash mobilized by wind and rain can further contaminate surface water and groundwater (*44*–*46*), creating persistent risks, and become a concern for residents returning to standing homes (*47*). Real-time, chemically resolved measurements, such as those presented here, enable tracking of these processes and are essential for constraining exposure and guiding postfire remediation yet remain rare in fire-prone airsheds. Capturing the toxic metal burden and downwind chemical evolution of these plumes will require expanding the geographical coverage of high–time resolution, chemically speciated PM measurements, improving emission inventories that distinguish structural fuels from biomass burning, and advancing atmospheric models that capture their distinct emissions and atmospheric processing. This study provides an empirical foundation that future measurement and modeling efforts can build on.

As climate change and urban expansion drive more fires into cities (*48*), the question is not whether another urban firestorm will occur. What remains to be seen is what we will have learned from the previous ones and whether monitoring and emergency response systems can adapt fast enough to characterize these emissions and their exposure risk. This study demonstrates that real-time, chemically resolved measurements can track the rapid evolution of urban fire emissions in ways that conventional monitoring cannot. Millions of residents faced an acute toxic metal burden with unknown health consequences. Understanding and ultimately mitigating these health risks must become a priority as such events grow more frequent and severe.

## MATERIALS AND METHODS

Descriptions of the ASCENT monitoring sites, trace element and MODULAIR PM_2.5_ measurements and analyses, fire enhancement ratio calculations, STILT atmospheric dispersion modeling, source apportionment analysis, and daily Pb population exposure estimation can be found in the Supplementary Materials.

## Supplementary Materials

### This PDF file includes:

Supplementary Text Figs. S1 to S12 Tables S1 and S2 References
