# Supplementary material for: Fire at the boundary: Airborne toxic metals from the 2025 Los Angeles urban firestorm
Source: Sci Adv. 2026 Oct 28;12(44):eaei4455. doi: 10.1126/sciadv.aei4455 (PMC13715721; doi:10.1126/sciadv.aei4455)
Supplement: Supplementary file 1 [file sciadv.aei4455_sm.pdf]

Science Advances
AAAS

# Supplementary Materials for

## Fire at the boundary: Airborne toxic metals from the 2025 Los Angeles urban firestorm

Haroula D. Baliaka *et al.*

Corresponding author: Haroula D. Baliaka, haroula@caltech.edu; Nga L. Ng, ng@caltech.edu

*Sci. Adv.* **12**, eaei4455 (2026)
DOI: 10.1126/sciadv.aei4455

**This PDF file includes:**

Supplementary Text
Figs. S1 to S12
Tables S1 and S2
References

# S1. Los Angeles Basin Site Descriptions

## S1.1. Pico Rivera, Los Angeles County

The primary measurement site is located in Pico Rivera, Los Angeles County, CA, approximately 14 miles south of the Eaton Canyon fire origin. Under typical meteorological conditions, the site is positioned downwind of central Los Angeles. The Pico Rivera site is one of the 12 sites in the Atmospheric Science and Chemistry mEasurement NeTwork (ASCENT), the first real-time, chemically speciated $PM_{2.5}$ measurement network in the U.S. The ASCENT Pico Rivera site has been operational since July 2023 (*40*) and is co-located with a South Coast Air Quality Management District (SCAQMD) monitoring site (*23*).

## S1.2. Rubidoux, Riverside County

A second ASCENT/SCAQMD site in Rubidoux, Riverside County, CA (*23*) was used to characterize wildland fire smoke for comparison with the Eaton urban fire plume. In September 2024, the Airport fire burned 23,526 acres in the Cleveland National Forest (*49*), impacting air quality across Riverside, with the plume reaching the Rubidoux site. Unlike the Eaton fire, the Airport fire burned predominantly wildland vegetation with minimal structural involvement, providing a reference chemical signature for wildland fire smoke. Co-located hourly $PM_{2.5}$ concentrations at this site were also used in the spatial fusion model.

## S1.3. Joshua Tree, San Bernardino County

A third ASCENT site in Joshua Tree, San Bernardino County, CA (*23*) was used in the $PM_{2.5}$ spatial fusion model for bias correction, described in section S7. This site is located in the Mojave Desert, approximately 130 miles east of Los Angeles, and typically provides a regional background reference.

## S2. Measurements

### S2.1. Elemental composition (Xact 625i)

Trace element concentrations were measured at 1-hour resolution using the Sailbri Cooper Xact 625i Ambient Continuous Multi-Metal Monitor that quantifies trace elements via energy-dispersive X-ray fluorescence (EDXRF) analysis.

The Xact software reports a per-measurement combined standard uncertainty for each element, computed as the quadrature sum of contributions from spectral deconvolution (the dominant term), flow, sampling volume, deposit area, and calibration standards. Trace elements were retained for further analysis only if $\geq 25\%$ of observations exceeded the individual method detection limits (MDL). Elements with $>75\%$ of values below detection were excluded. These are the final trace elements used in this study: Si, S, Cl, K, Ca, Ti, Cr, Mn, Fe, Ni, Cu, Zn, As, Se, Ba, Pb, Br. Note that for Rubidoux, the list of elements is the same, excluding Ni.

Hourly time series of the elements listed above, together with their respective reported uncertainties, are shown for Pico Rivera and Rubidoux in Figures S1 and S2 and illustrate three key features: (1) the chemical differences between urban and wildland fire emissions, with elements such as Pb, Zn, Cu, and As being much more elevated at Pico Rivera compared to Rubidoux; (2) the substantial variability within each event, with concentrations fluctuating by more than an order of magnitude within hours as plume intensity and source contributions shifted; and (3) distinct trends for different element groups, reflecting the evolving mix of burning materials. These dynamics would be missed by conventional time-integrated filter sampling.

### S2.2. Black carbon concentration (Aethalometer)

The Magee Scientific AE33 Aethalometer was used to measure black carbon (BC) concentrations. Optical absorption was measured at wavelengths of 370, 470, 520, 590, 660, 880, and 950 nm at 1-min time resolution. Absorption at 880 nm was used to determine the BC mass concentrations, assuming a mass absorption cross-section coefficient of $7.77\ m^2 g^{-1}$. We compare BC levels between the two fire events (Figure S3) and additionally use BC concentrations at the Pico Rivera site as an external tracer for source apportionment (section S6).

### S2.3. $PM_{2.5}$ mass concentration (MODULAIR)

Continuous $PM_{2.5}$ mass concentrations were measured using QuantAQ MODULAIR sensors, co-located at the three ASCENT sites. The MODULAIR sensors rely on light-scattering measurements that can be biased high during fog events when water droplets are counted as particles. Raw $PM_{2.5}$ measurements were corrected for fog interference following the method of Hagan and McClosky (*50*). The corrected $PM_{2.5}$ data were used for all subsequent analyses, including Pb/$PM_{2.5}$ ratio calculations described in section S7 and in Figure S4, where we compare $PM_{2.5}$ alongside the summed trace-element concentration at Pico Rivera during the Eaton fire.

### S2.4. Study periods

- Eaton fire: Measurements at Pico Rivera, January 5–17, 2025 (including fire plume period); September 2023–December 2024 (background period for source apportionment and baseline comparisons, excluding the 4th of July and New Year's Eve firework influence).
- Airport fire: Measurements at Rubidoux, September 2–12 2024 (including fire plume period).
- Structural vs. wildland fuel comparison: The Eaton fire destroyed 9,418 structures across 14,021 acres (*6*), with a structure density of 0.67 structures per acre. In contrast, the Airport fire burned 23,526 acres with only 160 structures lost (*49*) (0.0068 structures per acre). The 100-fold increase in structural density highlights the fundamental difference in fuel composition between the two events.
- Sandy fire (*51*): Measurements were collected at Pico Rivera during May 19–21, 2026. Although this fire was substantially smaller than the Airport and Eaton fires, its plume was detected at the site and provides an independent wildland-dominated event for evaluating the reproducibility of the K/Pb relationship (Figure S5).

## S3. Fire Enhancement Ratio Calculation

To quantify elemental enrichment during fire events, we calculated fire enhancement ratios by comparing mean elemental concentrations across distinct periods at each site.

For Pico Rivera, we define:

- *Pre-fire background*: January 5, 00:00 to January 7, 23:59, 2025 (this period represents typical ambient conditions before fire influence).

- *Fire plume* period: January 8, 10:00 to January 11, 10:00, 2025 (captures peak smoke impact from the 2025 Eaton fire).

For Rubidoux, we define analogous study periods:

- *Pre-fire background*: September 2, 00:00 to September 4, 00:00, 2024 (this represents typical ambient conditions before fire influence).

- *Fire plume* period: September 7, 20:00 to September 10, 12:00, 2024 (captures peak smoke impact at the Rubidoux site from the 2024 Airport fire).

For each element measured by the Xact 625i, mean concentrations were calculated for each period. Fire enhancement ratios were computed as the ratio of the *fire plume* period mean to the *pre-fire background* mean. These ratios quantify how much each element was elevated during active smoke impact compared to background conditions, enabling direct comparison of the chemical fingerprints between the urban firestorm (Eaton) and the wildland fire (Airport fire). Fire enhancement ratios greater than 1 indicate enrichment during the fire period, with higher values reflecting greater fire-related contributions.

## S4. Airborne Pb in Los Angeles

### S4.1. $PM_{2.5}$ Pb concentrations during the Eaton fire compared to background levels

Hourly $PM_{2.5}$ Pb concentrations were measured at the ASCENT Pico Rivera site. During the background period (September 2023–December 2024), hourly Pb averaged ~0.6 ng $m^{-3}$. The background reference pattern in Figure S6 (shaded, $\pm 1\sigma$) was computed using the mean diurnal Pb concentrations by day of the week from the background period.

### S4.2. Historical trends in ambient Pb

Daily Pb concentrations in total suspended particles (TSP) were obtained from the U.S. EPA Air Quality System (AQS) for the Los Angeles–North Main Street monitoring station (AQS Site ID:

06-037-1103), which provides one of the longest continuous records of ambient Pb in the Los Angeles Basin. TSP samples are collected on filters every sixth day and analyzed gravimetrically. Monthly averages were computed from all valid daily measurements within each month.

This record illustrates the substantial improvement in ambient Pb levels over four decades, from routine concentrations exceeding 1 $\mu$g m$^{-3}$ in the early 1980s to below 0.02 $\mu$g m$^{-3}$ in recent years (Figure S7). Although $PM_{2.5}$ Pb captures only the fine fraction of total suspended Pb, the highest 24-hour $PM_{2.5}$ Pb concentration measured at Pico Rivera during the Eaton fire (0.12 $\mu$g m$^{-3}$) exceeded typical modern TSP levels by more than an order of magnitude, underscoring the intensity of short-duration exposures that urban fire events can produce.

## S5. Stochastic Time-Inverted Lagrangian Transport (STILT) Model

We generated trajectories and footprints from the Stochastic Time-Inverted Lagrangian Transport (STILT) model (*52*) driven by the 3 km High-Resolution Rapid Refresh meteorological field (*53*) (Figure S8). Meanwhile, we estimated sectoral emissions using the 3 km total $PM_{2.5}$ emissions from the Regional ABI and VIIRS fire Emissions (RAVE) fire inventory (*54*), along with sectoral fractions based on fuel loads from the UT-Austin inventory (*21*). Sectoral fractions were assigned to two spatial domains: (1) urban burning and (2) wildland burning. The final step was to convolve STILT-HRRR footprints with estimated sectoral emissions (urban vs. wildfire). When trajectories or footprints (blue lines in Figure S8) overlapped substantially with the burned urban area of Altadena (dark brown in Figure S8), the resulting urban fraction was elevated, as shown by the teal shading in Figure 2A.

To evaluate the sensitivity of the plume source apportionment to meteorological inputs, we repeated the STILT back-trajectory analysis using an alternative meteorological field: HRRR at 3-km resolution coupled with a 1-km Large Eddy Simulation (LES), provided by Kay Suselj (*55*). LES was available only for the first two days of the fire (January 7–8, 2025). Figure S9 compares the urban and wildland source fractions derived from the two meteorological fields. Both configurations show broad agreement in the partitioning between urban and wildland plume contributions, indicating that the transport estimates presented in Figure 2A are not highly sensitive to the choice of meteorological resolution. Quantifying formal transport uncertainties remains an open challenge for atmospheric

dispersion models, given limited wind observations during the fires; the consistency between these two independent meteorological fields provides confidence in the source attribution results. The HRRR 3-km configuration was used in the main manuscript, given its longer temporal coverage.

Beyond the uncertainty in the meteorological driving fields discussed above, STILT footprints carry additional uncertainties associated with modeled winds, planetary boundary layer (PBL) height, deep convection, plume rise, and random error arising from finite particle statistics (*56–58*). Within the Lagrangian transport modeling literature, wind and PBL uncertainties are generally expected to dominate this budget (*55*). During this event, the strong and well-defined Santa Ana flow constrains the transport direction relatively tightly, while buoyancy-driven terms such as deep convection and plume rise are expected to contribute comparatively little. Lastly, these STILT-derived exposure fractions are not used quantitatively; they serve to support one of our main conclusions that lower K/Pb ratios are associated with urban rather than wildland fire smoke. A previous study found that the Pb emission factor for wildland fuels is one to two orders of magnitude lower than for structure or vehicle combustion (*25*). Because this difference in source emission strength far exceeds any plausible STILT transport error, the qualitative urban-versus-wildland distinction is robust to the transport uncertainties discussed above.

## S6. Source Apportionment

Source apportionment was conducted using the Positive Matrix Factorization (PMF) Evaluation Toolkit (PET v3.08) (*59, 60*) on hourly concentrations of the full retained element suite defined in section S2.1 for the period of January 4, 16:00 to January 17, 23:00, 2025 (local time), encompassing pre-plume, plume, and post-plume conditions. PMF is a receptor-based model widely used for source identification that decomposes measured concentrations into source profiles and time-varying contributions without requiring prior knowledge of source compositions.

Hourly-averaged elemental concentrations were prepared for PMF analysis following standard quality assurance procedures (*61*):

- Below-detection-limit treatment: For concentrations below the MDL, values were replaced with MDL/2 and their equivalent error was set to 5/6 MDL, following EPA guidance (*62*).

- Above-detection-limit: For concentrations above the MDL, errors were calculated as (*63*):

$$\sigma = \sqrt{\mathrm{MDL}^2 + \mathrm{unc}^2} \tag{S1}$$

  where unc is the reported uncertainty for each element reported by the instrument.

- Elements with signal-to-noise ratios between 0.2 and 2 were categorized as "weak" and down-weighted by a factor of 2; elements with signal-to-noise ratios <0.2 were excluded.

A five-factor solution was selected through several complementary diagnostics. The number of factors was determined by examining $Q/Q_{\mathrm{exp}}$ as a function of the number of factors, together with the distribution of scaled residuals and the physical interpretability of the resulting profiles (Figure S10). Five factors minimized the unexplained variance while yielding chemically distinct and physically meaningful sources. Solutions with fewer factors merged distinct sources, whereas additional factors split existing sources without improving the model fit. Scaled residuals for all species fell within ±3, indicating that the model reproduced the observed concentrations without systematic bias. Rotational ambiguity was assessed by varying `FPEAK` from $-1$ to $+1$. An `FPEAK` value of 0 was selected because it produced the most physically interpretable and stable factor profiles (Figure S10B).

The optimal solution was also determined after exploring diurnal trends of each factor, known-tracer signatures of factor profiles, and correlations with external tracers (Figure S11), like the BC measurements from a co-located aethalometer. We also compared with background PMF results from the same site. Specifically, PMF was applied to the full yearly dataset (September 2023–December 2024) and the mean diurnal profile for each factor was computed by day of the week. These background reference patterns are shown in the left panels of Figure 3 (shaded, $\pm 1\sigma$), capturing typical weekday–weekend variability and enabling direct comparison between fire-period contributions and expected background levels for each source. Note that the background contributions of the *urban materials* and *halogenated source* are not caused by fire combustion; in this context, the *urban materials* factor reflects resuspension and/or industrial emissions of trace metals (Pb, Zn, etc.), while the high Cl signature in the background *halogenated source* is associated with sea salt and local anthropogenic Cl sources. We use the same factor names for easy comparison between typical background concentrations and sources related to the fire.

## S7. Daily Pb Mapping and Exposure

### S7.1. $PM_{2.5}$ spatial fusion model

Daily 24-hour average $PM_{2.5}$ concentrations were estimated at 1-km spatial resolution across the Los Angeles Basin using a spatial machine-learning fusion approach that applies random forest regression to predict surface $PM_{2.5}$ (*33*). The method integrates official permanent and temporary regulatory monitors, low-cost sensor networks, satellite aerosol optical depth (AOD) retrievals, meteorological variables, and NOAA smoke forecast model output. The model was retrained for the Los Angeles basin over a 60-day window (December 2024–January 2025). The domain contains over 1,000 PurpleAir sensors and 41 regulatory $PM_{2.5}$ monitoring stations. In addition, the model was trained on the regulatory monitoring stations, with 30% removed as a test dataset, randomly selected each day over the 60-day modeling window. Lastly, the raw spatial fusion output was bias-corrected using 24-hour mean $PM_{2.5}$ concentrations from co-located MODULAIR sensors (QuantAQ) at the Pico Rivera, Rubidoux, and Joshua Tree ASCENT sites.

### S7.2. Pb concentration estimation

Daily Pb concentrations were estimated by applying Pb/$PM_{2.5}$ concentration ratios to the bias-corrected $PM_{2.5}$ fields. For smoke-impacted areas (identified using daily smoke plume extents from the NOAA Hazard Mapping System, HMS), we applied the daily Pb/$PM_{2.5}$ ratio calculated from Xact 625i Pb and MODULAIR $PM_{2.5}$ at the Pico Rivera site. For areas outside the HMS smoke plume boundary, we applied the mean Pb/$PM_{2.5}$ ratio measured during the pre-fire background period (January 1–6, 2025). Model performance against co-located observations is evaluated in section S7.4 (Figure S12).

### S7.3. Population exposure estimation

These daily Pb concentration maps were intersected with census tract-level population data from the U.S. Census Bureau 2019–2023 American Community Survey 5-Year Estimates to estimate outdoor exposure. For each day, we calculated the number of residents exposed to Pb concentrations exceeding various thresholds (e.g., 10, 25, 50, 100 ng $m^{-3}$). These estimates represent potential outdoor exposure and do not account for time-activity patterns or indoor air quality.

### S7.4. Sensitivity of exposure estimates

Because daily Pb was estimated by applying a day-specific Pb/$PM_{2.5}$ ratio measured at a single site (Pico Rivera) to the basin-wide $PM_{2.5}$ field (section S7.2), the exposure estimates carry two distinct uncertainties, which we bounded using the co-located Xact observations at the three ASCENT sites (Figure S12).

First, to quantify the uncertainty in Pb at the site where the ratio is applied, we computed the ratio of observed (by Xact 625i) to modeled Pb at Pico Rivera for each day of the fire period (January 8–11, 2025). This factor ranges from 0.71 to 1.49. The model underestimates observed Pb at Pico Rivera on the highest-concentration days (e.g., the January 9 peak, where the model predicts 83 ng $m^{-3}$ against an observed 124 ng $m^{-3}$). We propagated this range to the exposure estimates by scaling the daily Pb field by 0.71 and 1.49; because a uniform scaling preserves the rank ordering of census tracts, this is equivalent to a vertical rescaling of the cumulative-exposure curves (shaded bands, Figure 4B).

Second, the model overestimates observed Pb at the Rubidoux and Joshua Tree sites on every fire day (Figure S12). These sites are farther from the urban source, where $PM_{2.5}$ is less influenced by the Pb-rich structural-fire plume and carries a lower Pb/$PM_{2.5}$ than that measured at Pico Rivera; applying the Pico Rivera ratio there leads to overestimation of Pb.

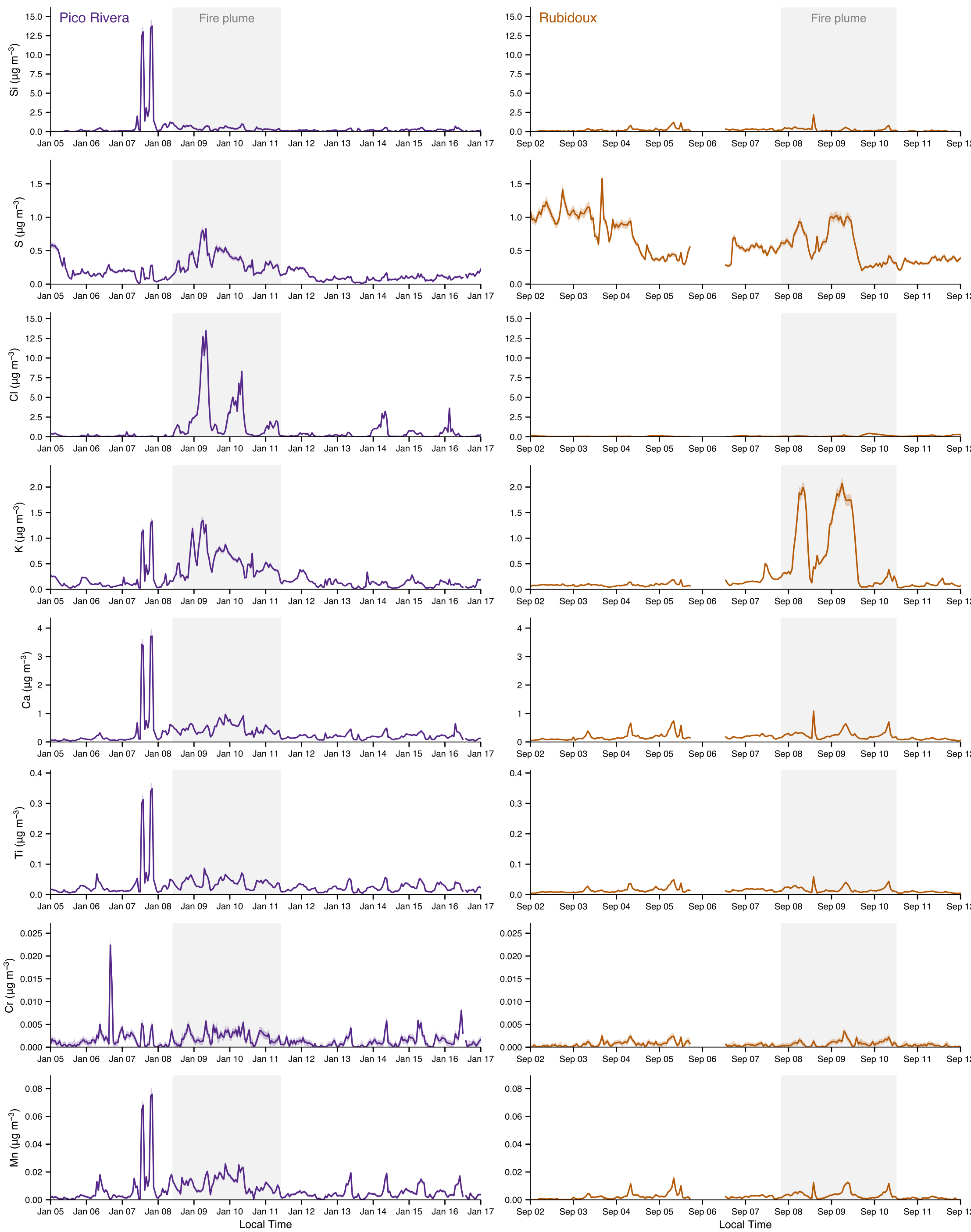


**Figure S1**: **Hourly elemental concentrations during fire events at Pico Rivera and Rubidoux (Part 1).** Time series of elements measured by the Xact 625i at the two ASCENT sites. Pico Rivera (purple, left column) captured the Eaton urban firestorm, while Rubidoux (orange, right column) captured the Airport wildland fire. Shaded bands denote the measurement uncertainty for each element. The gray shaded region indicates the *fire plume* period at each site as defined in section S3.

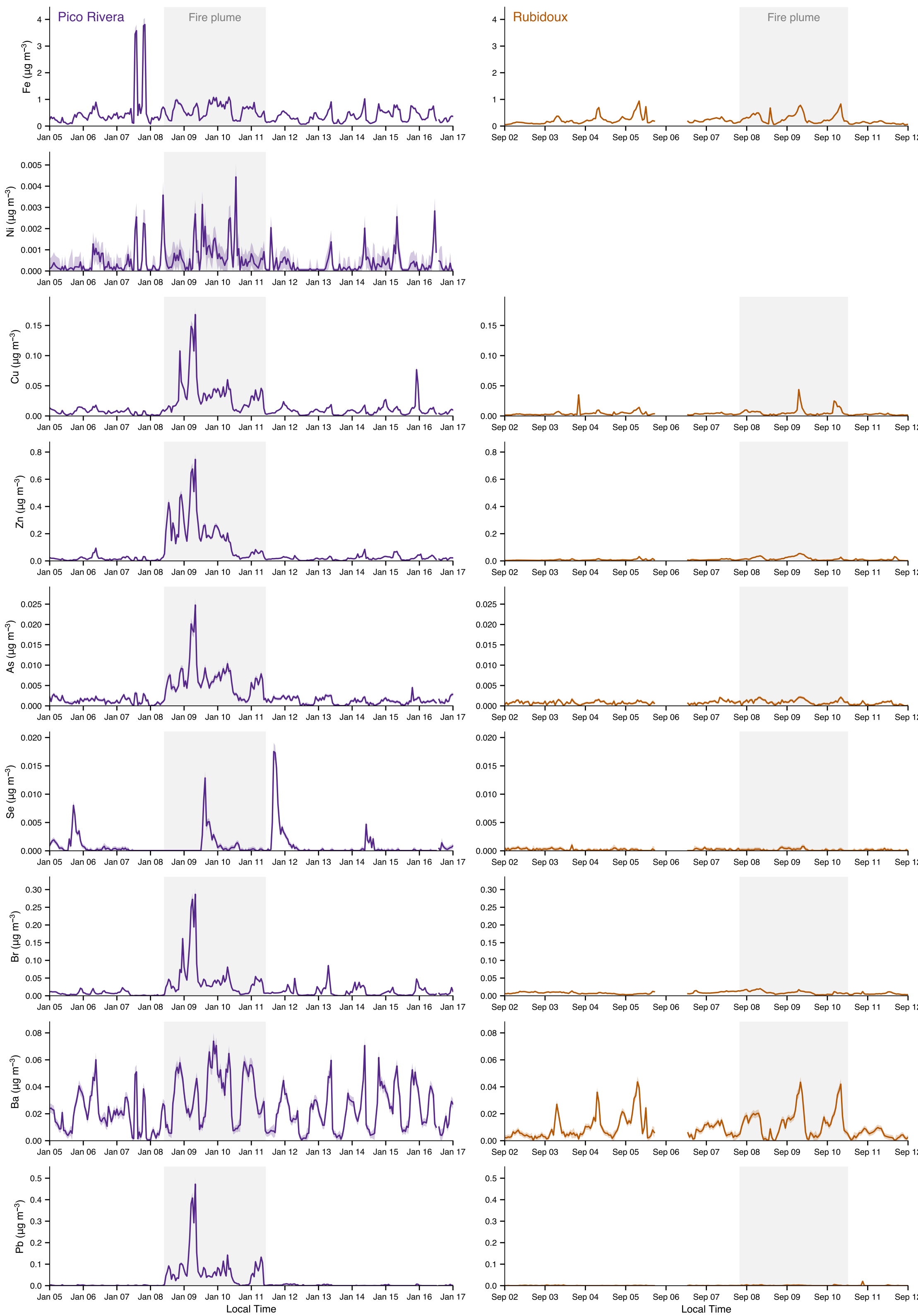


**Figure S2**: **Hourly elemental concentrations during fire events at Pico Rivera and Rubidoux (Part 2).** Continuation of Figure S1.

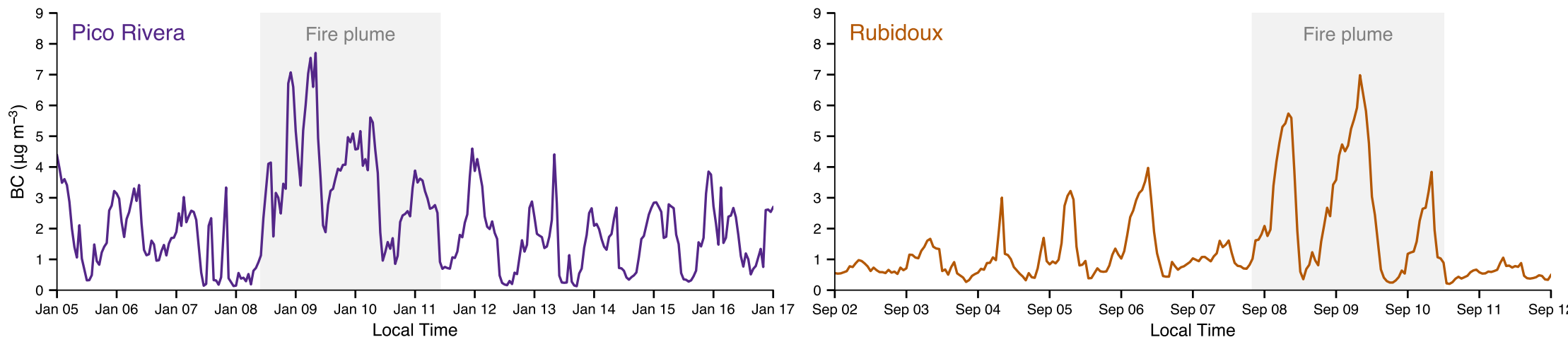


**Figure S3**: **Hourly black carbon (BC) concentrations during fire events at Pico Rivera and Rubidoux.** Time series of BC mass concentration measured by the aethalometer at the two ASCENT sites. Pico Rivera (purple, left) captured the Eaton urban firestorm, while Rubidoux (orange, right) captured the Airport wildland fire. The gray shaded region indicates the *fire plume* period at each site as defined in section S3.

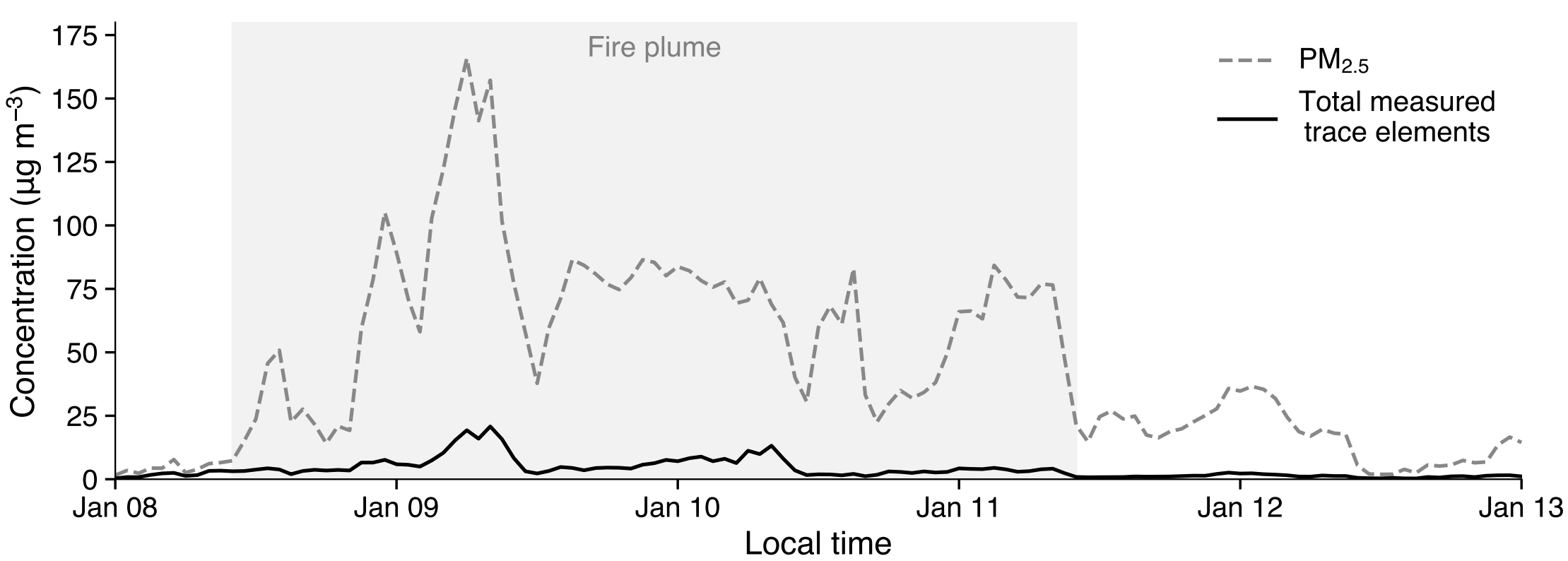


**Figure S4**: **Time series of $PM_{2.5}$ mass and total measured trace elements at Pico Rivera during the Eaton fire.** $PM_{2.5}$ mass concentration (gray dashed) and the sum of the retained suite of trace element concentrations (black solid) at Pico Rivera ($\mu$g m$^{-3}$; January 8–13, 2025). The gray shaded region indicates the fire plume period.

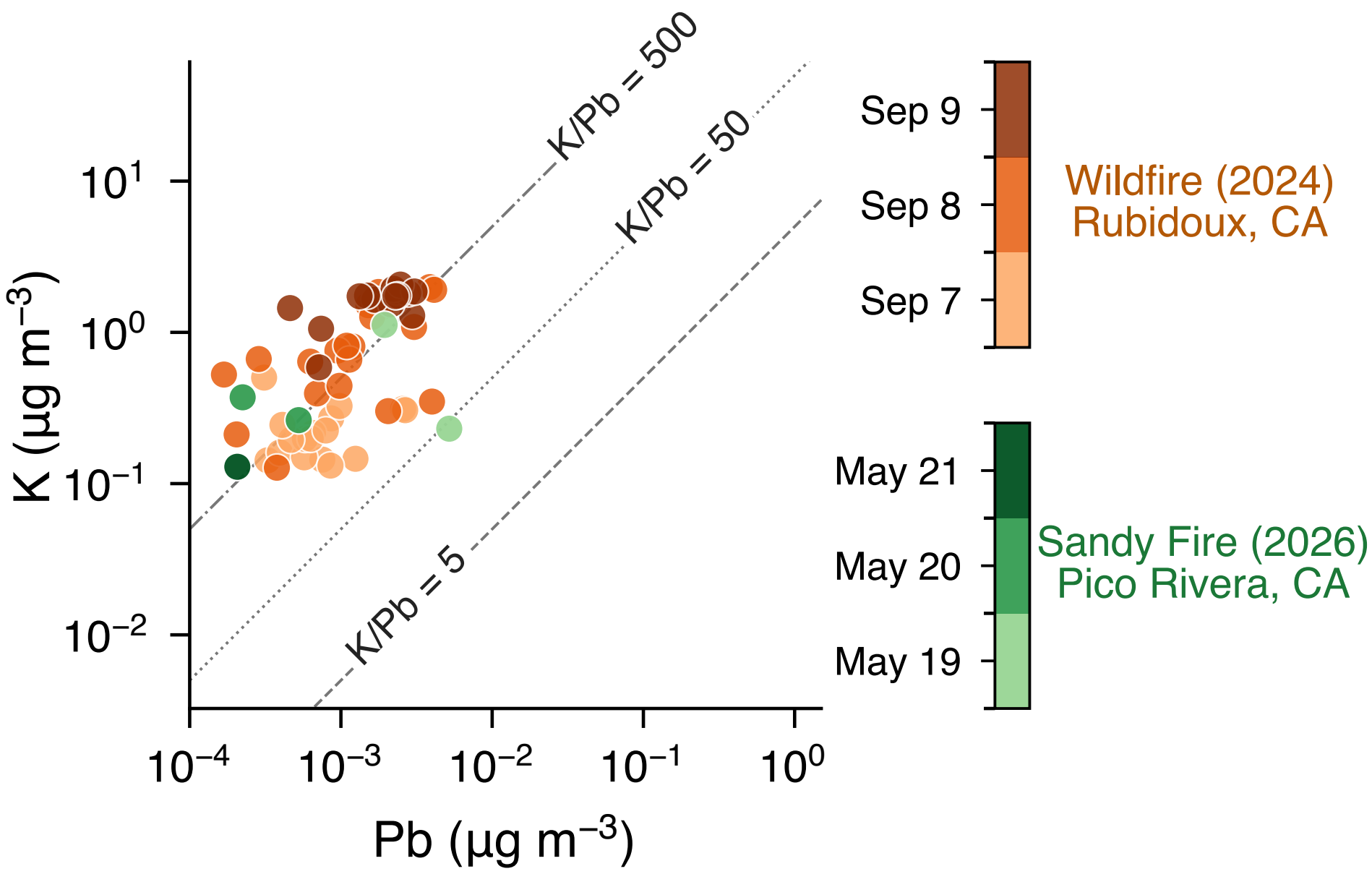


**Figure S5**: **Potassium-to-lead (K/Pb) ratios for two wildland-dominated fires.** K versus Pb concentrations ($\mu$g m$^{-3}$) measured at Rubidoux (September 7–9, 2024; oranges) during the Airport fire and at Pico Rivera (May 19–21, 2026; greens) during the Sandy fire. Dashed lines denote constant K/Pb ratios of 5, 50, and 500. The two plumes fall within a similar K/Pb range.

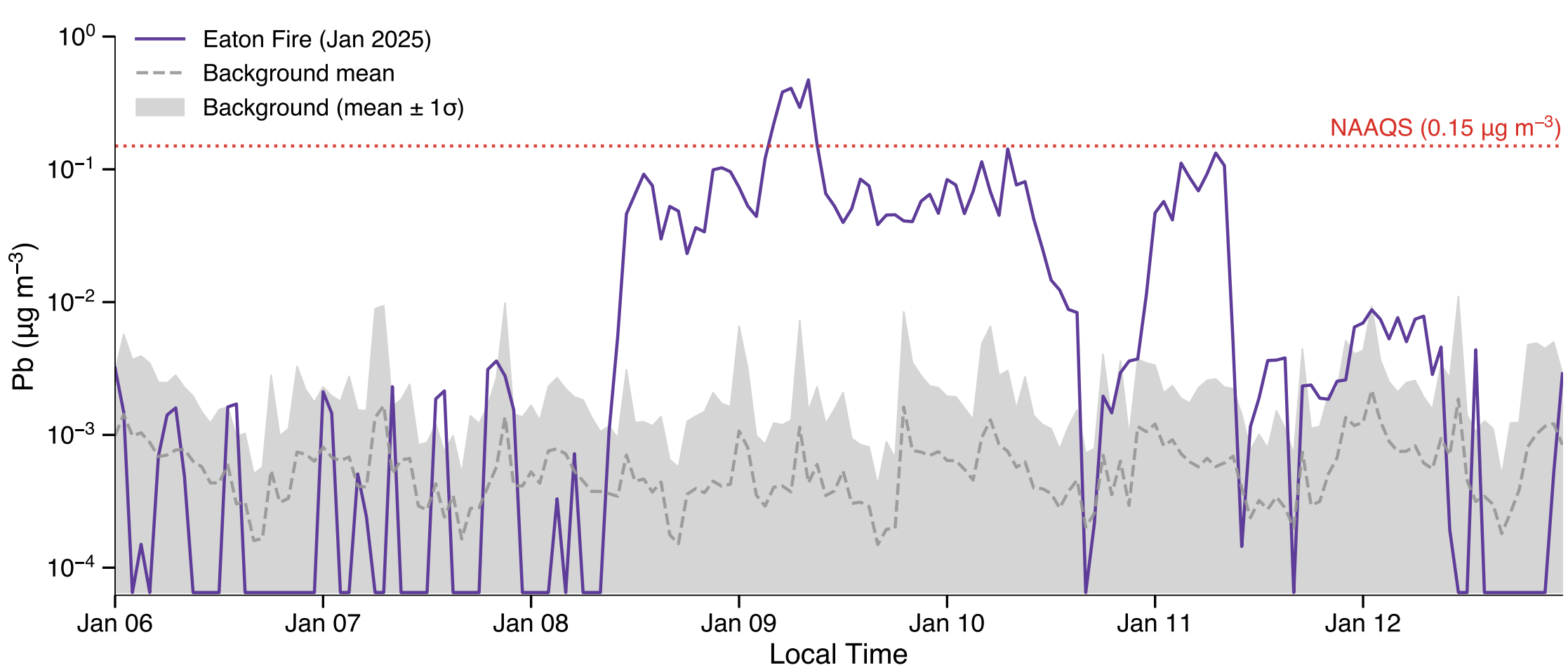


**Figure S6**: **Hourly Pb concentrations during the Eaton fire (*12*).** Pb at Pico Rivera (purple) compared to background diurnal pattern (gray dashed line shows background mean; gray shaded is mean $\pm$ 1$\sigma$). The red dotted line indicates the current National Ambient Air Quality Standard (NAAQS) for Pb (0.15 $\mu$g m$^{-3}$, 3-month rolling average).

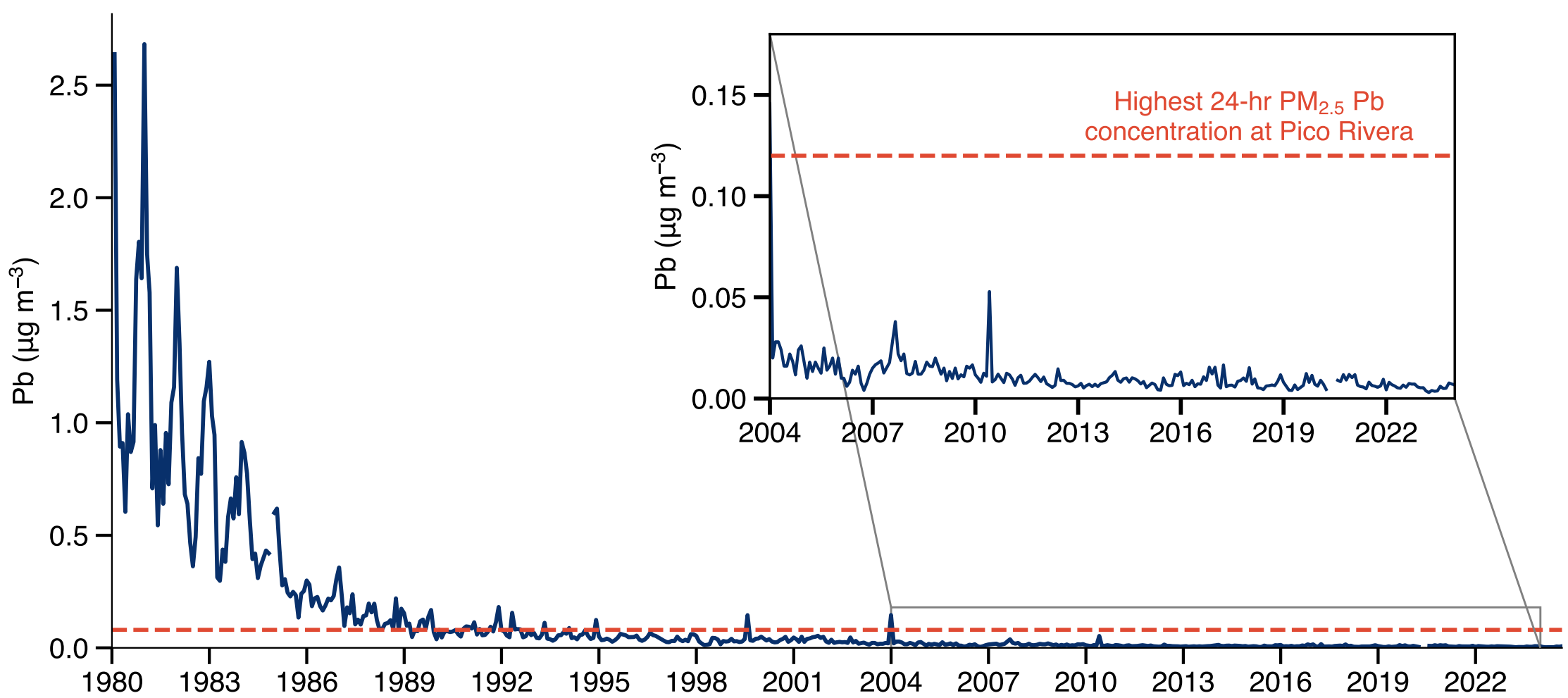


**Figure S7**: **Historical ambient Pb concentrations in downtown Los Angeles.** Monthly averaged total suspended particle (TSP) Pb concentrations measured at the Los Angeles–North Main Street monitoring station from 1980 to 2024 (U.S. EPA Air Quality System). The red dashed line marks the highest 24-hour average $PM_{2.5}$ Pb concentration (0.12 $\mu$g m$^{-3}$) measured at Pico Rivera during the January 2025 Eaton fire. Inset: expanded view of the 2004–2024 period. Although $PM_{2.5}$ lead (particles <2.5 $\mu$m) represents only a fraction of TSP Pb (all particle sizes), the Eaton fire peak exceeded typical modern TSP background levels by more than an order of magnitude.

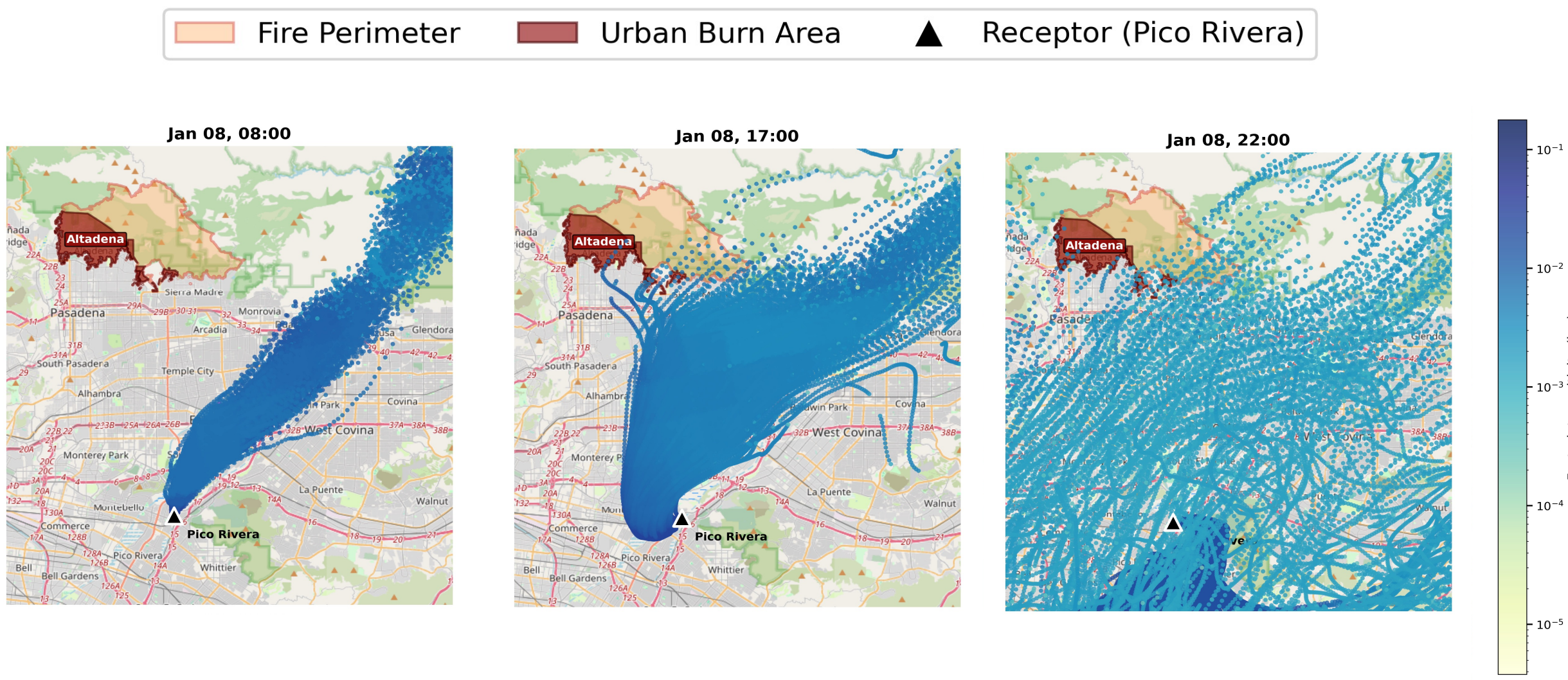


**Figure S8**: **STILT model footprints showing air parcel trajectories from the Pico Rivera receptor site during the Eaton fire.** Indicative examples of hourly back-trajectory footprints on January 8, 2025 at 08:00, 17:00, and 22:00 local time, showing the spatial extent of air masses sampled at the Pico Rivera site (black triangle). Color intensity indicates footprint sensitivity (log scale), with darker blue representing stronger influence on measured concentrations. The fire perimeter (orange) and urban burn area (dark red, Altadena) are overlaid. During the morning (8:00), trajectories do not intersect the fire. By 17:00, wind patterns have shifted and trajectories pass directly through the urban burn area and at 22:00, trajectories become more diffuse but continue to sample both urban and wildland portions of the fire.

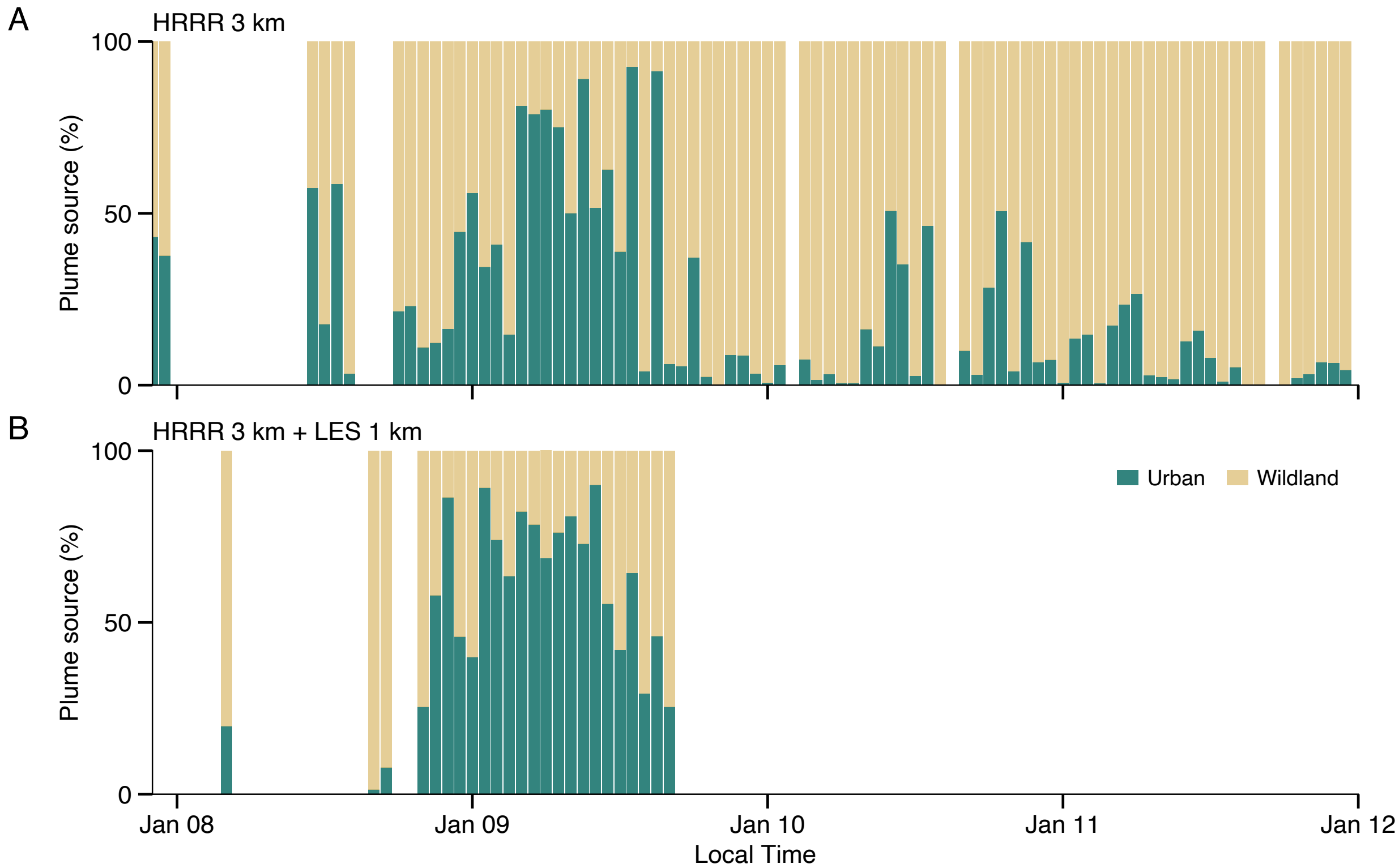


**Figure S9**: **Sensitivity of plume source apportionment to meteorological inputs.** Plume source origin (%) at the Pico Rivera receptor site estimated using the STILT atmospheric dispersion model with two meteorological fields: (**A**) HRRR at 3-km resolution and (**B**) HRRR at 3-km coupled with a 1-km Large Eddy Simulation (LES). The LES meteorology is available only for the first two days of the fire. Despite differences in spatial resolution and turbulence representation, both configurations yield broadly consistent urban-to-wildland source fractions, supporting the robustness of the transport estimates used in the main analysis (Figure 2A).

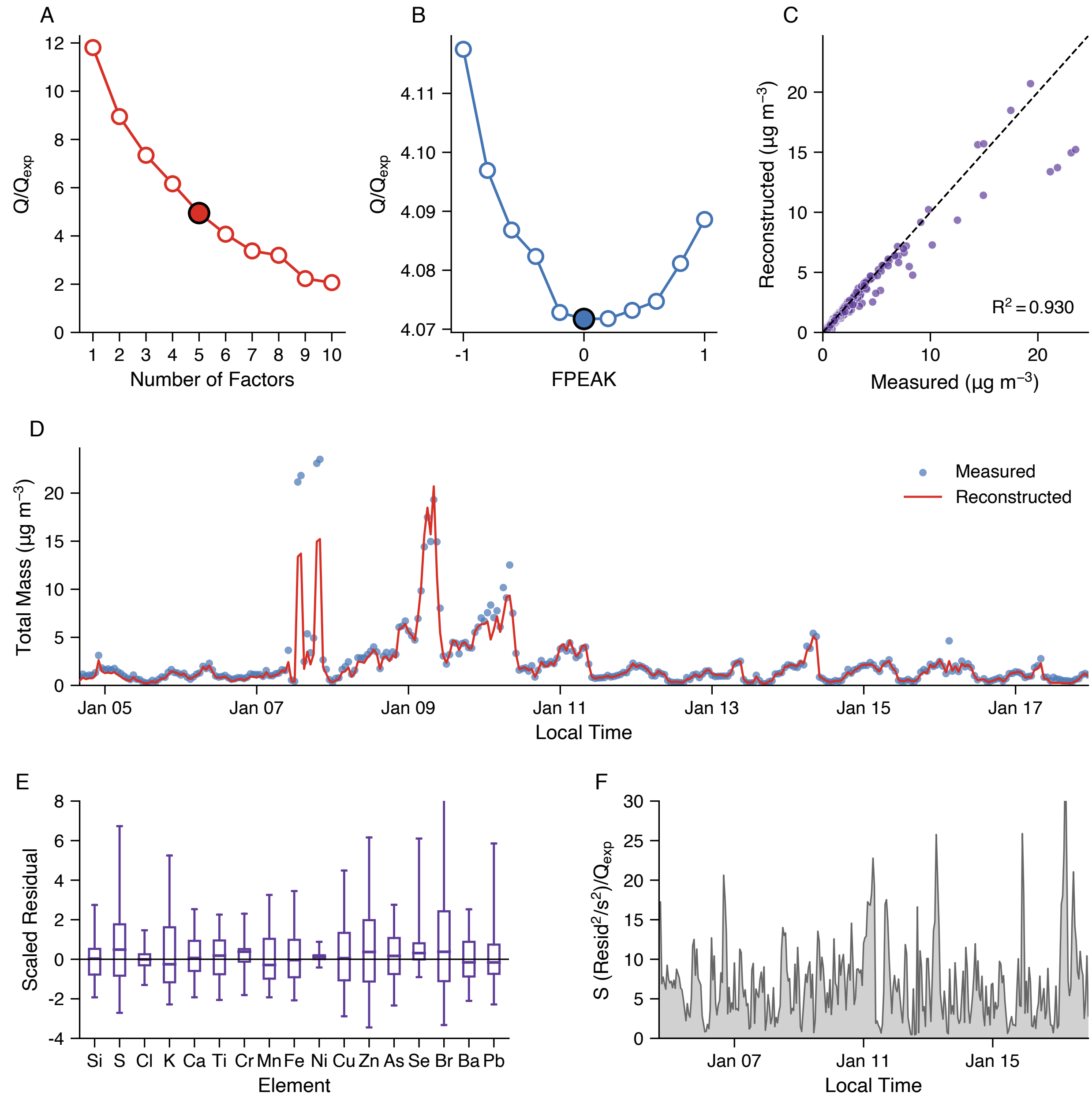


**Figure S10**: **PMF diagnostic summary for the five-factor solution.** (**A**) $Q/Q_{exp}$ versus number of factors; the five-factor solution (filled circle) was selected. (**B**) $Q/Q_{exp}$ versus FPEAK; FPEAK = 0 (filled circle) was selected. (**C**) Measured versus reconstructed total mass ($R^2$ = 0.93); dashed line indicates the 1:1 relationship. (**D**) Time series of measured (points) and reconstructed (line) total mass. (**E**) Scaled residuals by element shown as box plots (median, interquartile range, and whiskers). (**F**) $Q/Q_{exp}$ contribution for each time step.

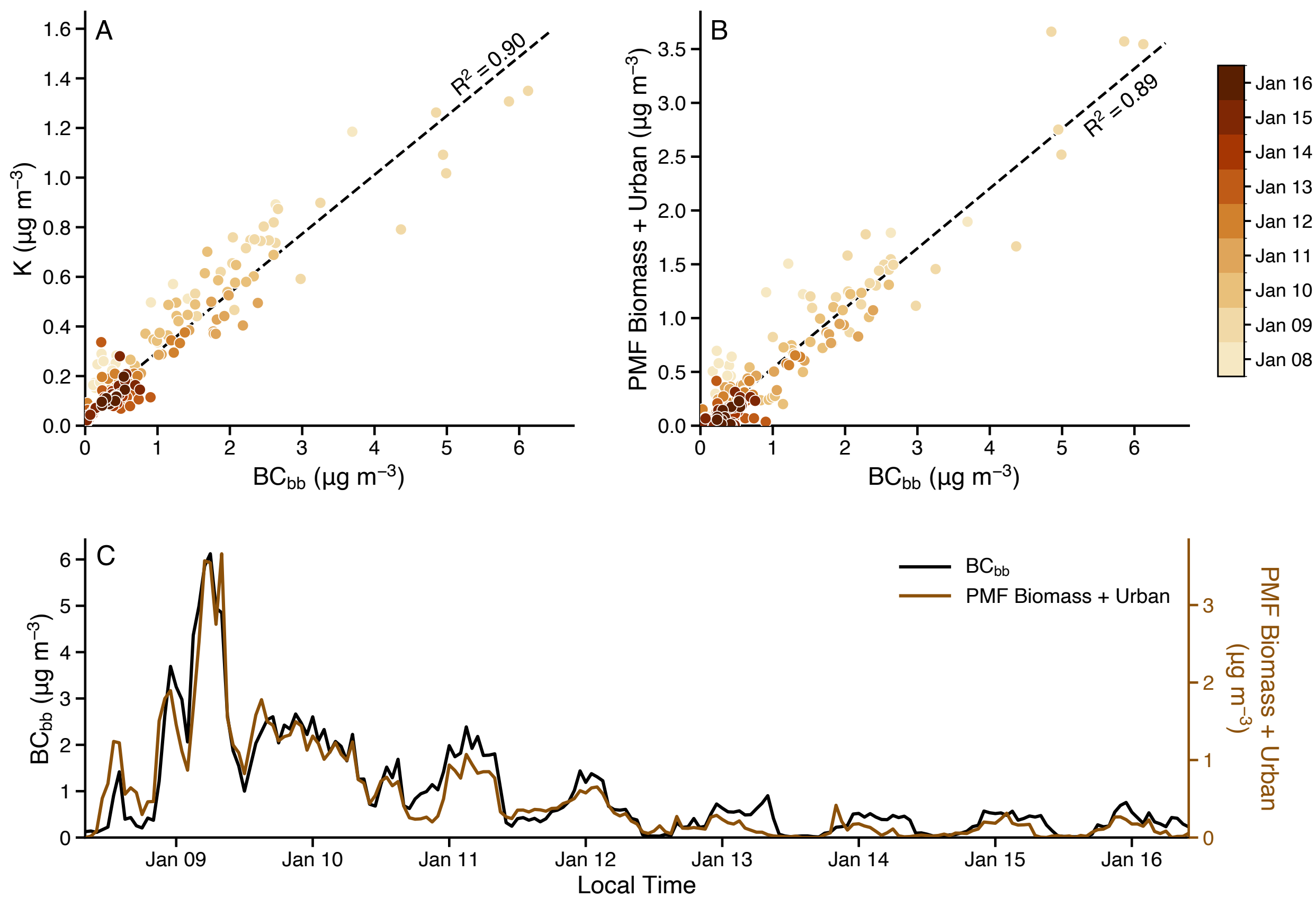


**Figure S11**: **Validation of PMF-resolved fire sources against independent black carbon measurements.** (**A**) Scatter plot of potassium (K) versus aethalometer-derived biomass burning black carbon ($BC_{bb}$) colored by date, showing strong correlation ($R^2 = 0.9$) consistent with K as a biomass burning tracer. (**B**) Scatter plot of combined PMF *biomass burning* and *urban materials* source contributions versus $BC_{bb}$. (**C**) Time series of $BC_{bb}$ (black) and PMF *biomass burning* and *urban materials* (brown) at the Pico Rivera site during January 8–16, 2025. The strong correlations between PMF-resolved sources and $BC_{bb}$ provide independent validation of the source apportionment results. Dashed lines in (A) and (B) represent linear regression fits.

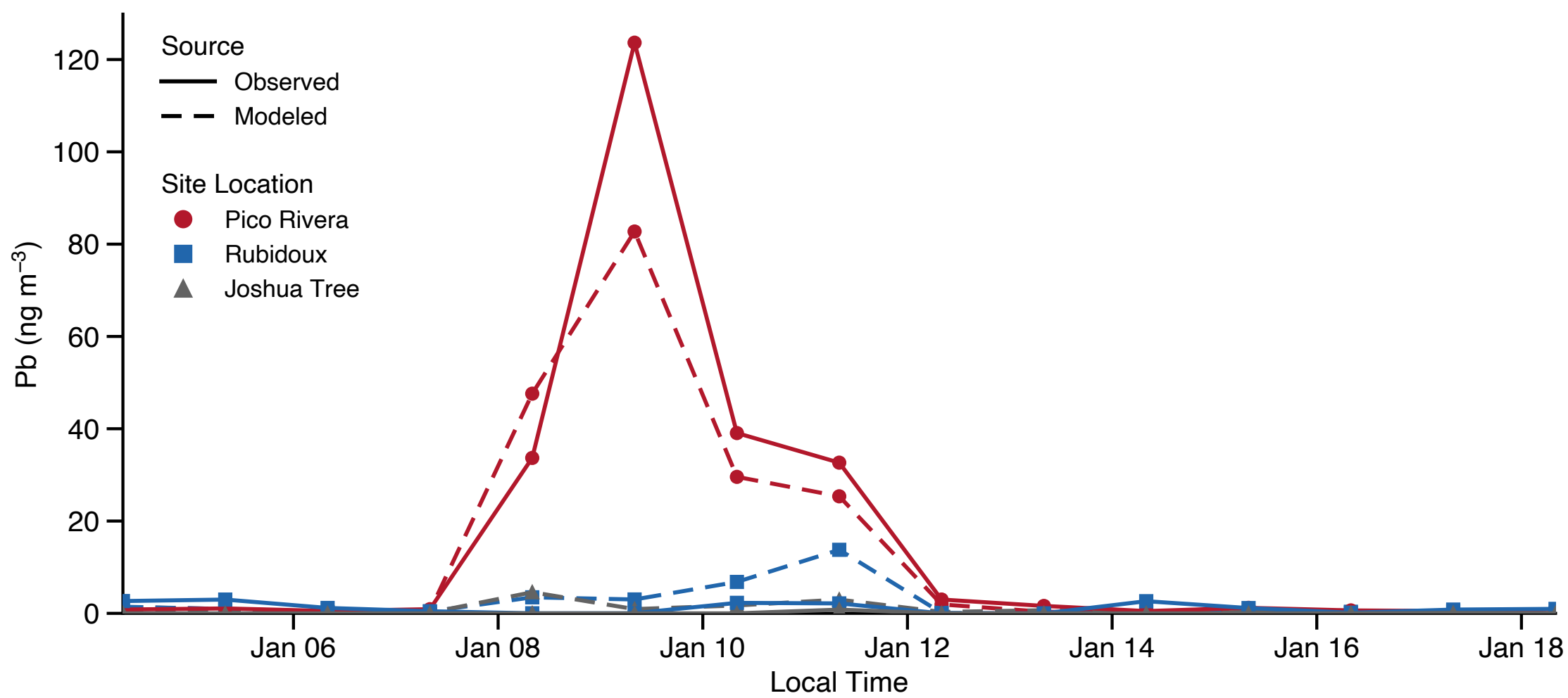


**Figure S12**: **Daily average Pb concentrations at three ASCENT monitoring sites during the January 2025 Los Angeles fires.** Observed (solid lines; Xact 625i) and modeled (dashed lines; spatial fusion model) 24-hour average $PM_{2.5}$ Pb concentrations at Pico Rivera (red), Rubidoux (blue), and Joshua Tree (gray). Modeled concentrations were obtained from the 1-km spatial fusion model explained in section S7.1. (*33*).

**Table S1**: **Elemental and $PM_{2.5}$ concentrations and fire enhancement ratios at Pico Rivera during the Eaton fire.** Columns are peak and mean concentrations during the fire plume period (January 8, 10:00 to January 11, 10:00, 2025) and mean background concentrations during the pre-fire period (January 5, 00:00 to January 7, 23:59, 2025), as defined in section S3. The fire enhancement ratio is the plume-period mean divided by the pre-fire background mean; values greater than 1 indicate enrichment during the fire.

| Species | Peak ($\mu g\, m^{-3}$) | Plume mean ($\mu g\, m^{-3}$) | Background mean ($\mu g\, m^{-3}$) | Enhancement |
|---|---|---|---|---|
| K | 1.4 | 0.55 | 0.18 | 2.99 |
| Mn | 0.026 | 0.011 | 0.0076 | 1.51 |
| Si | 1.1 | 0.40 | 1.0 | 0.40 |
| Ba | 0.074 | 0.035 | 0.020 | 1.70 |
| Ca | 0.97 | 0.47 | 0.33 | 1.41 |
| Fe | 1.1 | 0.59 | 0.54 | 1.10 |
| Ti | 0.086 | 0.038 | 0.038 | 1.02 |
| Se | 0.013 | 0.00089 | 0.00078 | 1.15 |
| S | 0.83 | 0.35 | 0.20 | 1.72 |
| As | 0.025 | 0.0063 | 0.0014 | 4.59 |
| Cu | 0.17 | 0.037 | 0.0072 | 5.08 |
| Br | 0.29 | 0.049 | 0.0071 | 6.86 |
| Zn | 0.75 | 0.19 | 0.017 | 11.42 |
| Cl | 13 | 2.4 | 0.10 | 22.88 |
| Pb | 0.47 | 0.075 | 0.00086 | 86.82 |
| $PM_{2.5}$ | 170 | 66 | 14 | 4.59 |

**Table S2**: **Elemental and $PM_{2.5}$ concentrations and fire enhancement ratios at Rubidoux during the Airport fire.** Columns are peak and mean concentrations during the fire plume period (September 7, 20:00 to September 10, 12:00, 2024) and mean background concentrations during the pre-fire period (September 2, 00:00 to September 4, 00:00, 2024), as defined in section S3. The fire enhancement ratio is the plume-period mean divided by the pre-fire background mean; values greater than 1 indicate enrichment during the fire.

| Species | Peak ($\mu g\, m^{-3}$) | Plume mean ($\mu g\, m^{-3}$) | Background mean ($\mu g\, m^{-3}$) | Enhancement |
|---|---|---|---|---|
| K | 2.0 | 0.63 | 0.078 | 8.14 |
| Mn | 0.012 | 0.0034 | 0.00099 | 3.40 |
| Si | 2.2 | 0.25 | 0.092 | 2.70 |
| Ba | 0.042 | 0.012 | 0.0055 | 2.22 |
| Ca | 1.1 | 0.24 | 0.11 | 2.14 |
| Fe | 0.83 | 0.27 | 0.14 | 1.94 |
| Ti | 0.059 | 0.016 | 0.0093 | 1.73 |
| Se | 0.00077 | 0.00018 | 0.00029 | 0.64 |
| S | 1.0 | 0.55 | 1.0 | 0.54 |
| As | 0.0021 | 0.00096 | 0.00074 | 1.29 |
| Cu | 0.025 | 0.0054 | 0.0034 | 1.59 |
| Br | 0.020 | 0.0087 | 0.0089 | 0.98 |
| Zn | 0.049 | 0.016 | 0.0055 | 2.86 |
| Cl | 0.42 | 0.12 | 0.039 | 3.02 |
| Pb | 0.0054 | 0.0012 | 0.00080 | 1.56 |
| $PM_{2.5}$ | 180 | 52 | 14 | 3.85 |

## REFERENCES

1. V. C. Radeloff, D. P. Helmers, H. A. Kramer, M. H. Mockrin, P. M. Alexandre, A. Bar-Massada, V. Butsic, T. J. Hawbaker, S. Martinuzzi, A. D. Syphard, S. I. Stewart, Rapid growth of the US wildland-urban interface raises wildfire risk. *Proc. Natl. Acad. Sci. U.S.A.* **115**, 3314–3319 (2018).

2. K. Boaggio, S. D. LeDuc, R. B. Rice, P. F. Duffney, K. M. Foley, A. L. Holder, S. McDow, C. P. Weaver, Beyond particulate matter mass: Heightened levels of lead and other pollutants associated with destructive fire events in California. *Environ. Sci. Technol.* **56**, 14272–14283 (2022).

3. B. Rooney, Y. Wang, J. H. Jiang, B. Zhao, Z.-C. Zeng, J. H. Seinfeld, Air quality impact of the Northern California camp fire of november 2018. *Atmos. Chem. Phys.* **20**, 14597–14616 (2020).

4. W. D. Dresser, J. M. Silberstein, C. E. Reid, M. E. Vance, C. Wiedinmyer, M. P. Hannigan, J. A. de Gouw, Volatile organic compounds inside homes impacted by smoke from the marshall fire. *ACS EST Air* **2**, 4–12 (2025).

5. D. N. Farrant, C. Trauernicht, A. Kagawa-Viviani, T. W. Giambelluca, C. M. D'Antonio, On the anniversary of the Maui fires, a call for indigenous land care to mitigate future disasters. *Proc. Natl. Acad. Sci. U.S.A.* **121**, e2401045121 (2024).

6. CAL FIRE, Eaton Fire (2025); https://fire.ca.gov/incidents/2025/1/7/eaton-fire.

7. CAL FIRE, Palisades Fire (2025); https://fire.ca.gov/incidents/2025/1/7/palisades-fire.

8. K. Guirguis, B. Hatchett, R. Clemesha, R. Aguilera, A. Gershunov, I. Campbell, D. Cayan, M. Merrifield, Compound atmospheric drivers of the catastrophic 2025 Los Angeles urban firestorm. *npj Nat. Hazards* **2**, 103 (2025).

9. S. Kerber, D. Alkonis, D. Gorham, G. P. Horn, "Southern California fires timeline report" (Report, UL Research Institutes, 2025); https://doi.org/10.60752/102376.29905157.v1.

10. CAL FIRE, Statistics (2026); https://fire.ca.gov/our-impact/statistics.

11. C. Schollaert, R. Connolly, L. Cushing, M. Jerrett, T. Liu, M. Marlier, Air quality impacts of the january 2025 Los Angeles wildfires: Insights from public data sources. *Environ. Sci. Technol. Lett.* **12**, 911–917 (2025).

12. H. D. Baliaka, R. X. Ward, R. Bahreini, A. M. Dillner, A. G. Russell, J. H. Seinfeld, R. C. Flagan, P. O. Wennberg, N. L. Ng, Notes from the field: Elevated atmospheric lead levels during the Los Angeles urban fires—California, January 2025. *MMWR. Morb. Mortal. Wkly. Rep.* **74**, 69–71 (2025).

13. R. Aguilera, T. Corringham, A. Gershunov, T. Benmarhnia, Wildfire smoke impacts respiratory health more than fine particles from other sources: Observational evidence from Southern California. *Nat. Commun.* **12**, 1493 (2021).

14. Y. Liang, D. Sengupta, M. J. Campmier, D. M. Lunderberg, J. S. Apte, A. H. Goldstein, Wildfire smoke impacts on indoor air quality assessed using crowdsourced data in California. *Proc. Natl. Acad. Sci. U.S.A.* **118**, e2106478118 (2021).

15. M. Qiu, J. Li, C. F. Gould, R. Jing, M. Kelp, M. L. Childs, J. Wen, Y. Xie, M. Lin, M. V. Kiang, S. Heft-Neal, N. S. Diffenbaugh, M. Burke, Wildfire smoke exposure and mortality burden in the USA under climate change. *Nature* **647**, 935–943 (2025).

16. C. E. Reid, M. Brauer, F. H. Johnston, M. Jerrett, J. R. Balmes, C. T. Elliott, Critical review of health impacts of wildfire smoke exposure. *Environ. Health Perspect.* **124**, 1334–1343 (2016).

17. National Academies of Sciences, Engineering, and Medicine; Division on Earth and Life Studies; Board on Chemical Sciences and Technology; Committee on the Chemistry of Urban Wildfires, *The Chemistry of Fires at the Wildland-Urban Interface*, The National Academies Collection: Reports Funded by National Institutes of Health (National Academies Press (US), Washington (DC) (2022).

18. A. J. Pryor, D. E. Johnson, N. N. Jackson, Hazards of Smoke and Toxic Gases Produced in Urban Fires (1969).

19. T. Li, H. Chen, J. C. H. Fung, D. H. L. Chan, A. L. C. Yu, K. K. M. Leung, J. Z. Yu, Large presence of bromine and toxic metals in ambient fine particles from urban fires. *Atmos. Environ.* **295**, 119554 (2023).

20. W. Tang, C. Wiedinmyer, L. K. Emmons, A. L. Holder, J. H. Uhl, L. A. St. Denis, M. Cook, R. Abolafia-Rosenzweig, C. He, K. C. Barsanti, S. Leyk, J. T. Abatzoglou, J. K. Balch, H. Worden, P. F. Levelt, Emissions from burned structures in wildfires as significant yet unaccounted sources of US air pollution. *Nat. Commun.* **16**, 11443 (2025).

21. C. Schissel, M. Meyer, E. Thompson, Y. Kimura, S. Stokes, M. J. Kleeman, S. Raffuse, D. T. Allen, Fine spatial and temporal scale fuel and emissions inventories for the Eaton and Palisades Wildland Urban Interface (WUI) fires. *ACS EST Air* **3**, 156–164 (2026).

22. U.S. Census Bureau, B25034: Year Structure Built — American Community Survey Table (2024); https://data.census.gov/table/ACSDT5Y2024.B25034?q=Altadena+CDP,+California&t=Year+Structure+Built.

23. ASCENT | ASCENT, https://ascent.caltech.edu/.

24. W. Tang, L. K. Emmons, C. Wiedinmyer, D. B. Partha, Y. Huang, C. He, J. Zhang, K. C. Barsanti, B. Gaubert, D. S. Jo, J. Zhang, R. Buchholz, S. Tilmes, F. Vitt, C. Granier, H. M. Worden, P. F. Levelt, Disproportionately large impacts of wildland-urban interface fire emissions on global air quality and human health. *Sci. Adv.* **11**, eadr2616 (2025).

25. A. L. Holder, A. Ahmed, J. M. Vukovich, V. Rao, Hazardous air pollutant emissions estimates from wildfires in the wildland urban interface. *PNAS Nexus* **2**, pgad186 (2023).

26. M. O. Andreae, Soot carbon and excess fine potassium: Long-range transport of combustion-derived aerosols. *Science* **220**, 1148–1151 (1983).

27. R. K. Stevens, Sampling and analysis methods for use in source apportionment studies to determine impact of wood burning on fine particle mass. *Environ. Int.* **11**, 271–283 (1985).

28. M. H. Sowlat, S. Hasheminassab, B. Grant, P. Pakbin, J. Low, A. Polidori, Temporal shifts in sources and composition of particulate metals in an environmental justice community:

Impacts of continued emission reductions, COVID-19, and wildfires. *Atmos. Environ.* **336**, 120760 (2024).

29. D. M. Murphy, P. K. Hudson, D. J. Cziczo, S. Gallavardin, K. D. Froyd, M. V. Johnston, A. M. Middlebrook, M. S. Reinard, D. S. Thomson, T. Thornberry, A. S. Wexler, Distribution of lead in single atmospheric particles. *Atmos. Chem. Phys.* **7**, 3195–3210 (2007).

30. A. L. Wani, A. Ara, J. A. Usmani, Lead toxicity: A review. *Interdiscip. Toxicol.* **8**, 55–64 (2015).

31. US EPA, Center for Public Health and Environmental Assessment, Integrated Science Assessment (ISA) for Lead (Final Report); https://assessments.epa.gov/isa/document/&deid%3D359536 (2023).

32. T. I. Lidsky, J. S. Schneider, Lead neurotoxicity in children: Basic mechanisms and clinical correlates. *Brain* **126**, 5–19 (2003).

33. S. Raffuse, S. O'Neill, R. Schmidt, A model for rapid $PM_{2.5}$ exposure estimates in wildfire conditions using routinely available data: Rapidfire v0.1.3. *Geosci. Model Dev.* **17**, 381–397 (2024).

34. G. Pershagen, The carcinogenicity of arsenic. *Environ. Health Perspect.* **40**, 93–100 (1981).

35. OAR. US EPA, Initial List of Hazardous Air Pollutants with Modifications, https://epa.gov/haps/initial-list-hazardous-air-pollutants-modifications (2015).

36. A. L. Holder, A. P. Sullivan, Emissions, chemistry, and the environmental impacts of wildland fire. *Environ. Sci. Technol. Lett.* **11**, 903–907 (2024).

37. J. M. Silberstein, L. E. Mael, C. R. Frischmon, E. S. Rieves, E. R. Coffey, T. Das, W. Dresser, A. C. Hatch, J. Nath, H. O. Pliszka, C. E. Reid, M. E. Vance, C. Wiedinmyer, J. A. De Gouw, M. P. Hannigan, Residual impacts of a wildland urban interface fire on urban particulate matter and dust: A study from the Marshall Fire. *Air Qual. Atmos. Health* **16**, 1839–1850 (2023).

38. Y. Tao, T. C. VandenBoer, P. R. Veres, C. Warneke, J. A. de Gouw, R. J. Weber, M. Z. Markovic, Y. Zhao, K. R. Baker, J. T. Kelly, J. G. Murphy, C. J. Young, J. M. Roberts, Hydrogen chloride (HCl) at ground sites during CalNex 2010 and insight into its thermodynamic properties. *J. Geophys. Res. Atmos.* **127**, 1–16 (2022).

39. J. Ofodile, E. Y. Pfannerstill, C. Arata, S. E. Pusede, C. E. Ivey, A. H. Goldstein, Inequality in hazardous air pollutant emissions and concentrations measured over Los Angeles. *Environ. Sci. Technol.* **59**, 7588–7599 (2025).

40. R. X. Ward, H. D. Baliaka, B. C. Schulze, G. H. Kerr, J. D. Crounse, S. Hasheminassab, R. Bahreini, A. M. Dillner, A. Russell, N. L. Ng, P. O. Wennberg, R. C. Flagan, J. H. Seinfeld, Poorly quantified trends in ammonium nitrate remain critical to understand future urban aerosol control strategies. *Sci. Adv.* **11**, eadt8957 (2025).

41. C. J. Young, R. A. Washenfelder, P. M. Edwards, D. D. Parrish, J. B. Gilman, W. C. Kuster, L. H. Mielke, H. D. Osthoff, C. Tsai, O. Pikelnaya, J. Stutz, P. R. Veres, J. M. Roberts, S. Griffith, S. Dusanter, P. S. Stevens, J. Flynn, N. Grossberg, B. Lefer, J. S. Holloway, J. Peischl, T. B. Ryerson, E. L. Atlas, D. R. Blake, S. S. Brown, Chlorine as a primary radical: Evaluation of methods to understand its role in initiation of oxidative cycles. *Atmos. Chem. Phys.* **14**, 3427–3440 (2014).

42. D. S. Wang, L. Hildebrandt Ruiz, Chlorine-initiated oxidation of *n*-alkanes under high-NO*x* conditions: Insights into secondary organic aerosol composition and volatility using a FIGAERO–CIMS. *Atmos. Chem. Phys.* **18**, 15535–15553 (2018).

43. R. J. Yokelson, J. D. Crounse, P. F. DeCarlo, T. Karl, S. Urbanski, E. Atlas, T. Campos, Y. Shinozuka, V. Kapustin, A. D. Clarke, A. Weinheimer, D. J. Knapp, D. D. Montzka, J. Holloway, P. Weibring, F. Flocke, W. Zheng, D. Toohey, P. O. Wennberg, C. Wiedinmyer, L. Mauldin, A. Fried, D. Richter, J. Walega, J. L. Jimenez, K. Adachi, P. R. Buseck, S. R. Hall, R. Shetter, Emissions from biomass burning in the Yucatan. *Atmos. Chem. Phys.* **9**, 5785–5812 (2009).

44. C. R. Proctor, J. Lee, D. Yu, A. D. Shah, A. J. Whelton, Wildfire caused widespread drinking water distribution network contamination. *AWWA Wat. Sci.* **2**, e1183 (2020).

45. S. S. Schulze, E. C. Fischer, Prediction of water distribution system contamination based on wildfire burn severity in wildland urban interface communities. *ACS EST Water* **1**, 291–299 (2021).

46. L. J. Magliozzi, S. J. Matiasek, C. N. Alpers, J. A. Korak, D. McKnight, A. L. Foster, J. N. Ryan, D. A. Roth, P. Ku, M. T.-K. Tsui, A. T. Chow, J. P. Webster, Wildland–urban interface wildfire increases metal contributions to stormwater runoff in paradise, California. *Environ. Sci. Process. Impacts* **26**, 667–685 (2024).

47. M. Kleeman, C. Cappa, P. Green, Y. Zhou, M. Astaneh, S. Kant, Y. Zhu, M. Jerrett, Airborne Hexavalent Chromium Nanoparticles Detected Around Cleanup Zones for the 2025 Los Angeles Wildfires (2025); https://doi.org/10.21203/rs.3.rs-7401328/v1.

48. J. T. Abatzoglou, A. P. Williams, Impact of anthropogenic climate change on wildfire across Western US forests. *Proc. Natl. Acad. Sci. U.S.A.* **113**, 11770–11775 (2016).

49. CAL FIRE, Airport Fire (2024); https://fire.ca.gov/incidents/2024/9/9/airport-fire.

50. D. Hagan, D. McClosky, Identifying and Removing Data Records Influenced by Fog (2024); https://doi.org/10.5281/zenodo.10793534.

51. CAL FIRE, Sandy Fire, https://fire.ca.gov/incidents/2026/5/18/sandy-fire.

52. J. C. Lin, C. Gerbig, S. C. Wofsy, A. E. Andrews, B. C. Daube, K. J. Davis, C. A. Grainger, A near-field tool for simulating the upstream influence of atmospheric observations: The Stochastic Time-Inverted Lagrangian Transport (STILT) model. *J. Geophys. Res. Atmos.* **108**, 4493 (2003).

53. E. P. James, C. R. Alexander, D. C. Dowell, S. S. Weygandt, S. G. Benjamin, G. S. Manikin, J. M. Brown, J. B. Olson, M. Hu, T. G. Smirnova, T. Ladwig, J. S. Kenyon, D. D. Turner, The High-Resolution Rapid Refresh (HRRR): An Hourly Updating Convection-Allowing Forecast Model. Part II: Forecast Performance (2022); https://doi.org/10.1175/WAF-D-21-0130.1.

54. S. Kondragunta, D. Hanjun, Regional Hourly Advanced Baseline Imager (ABI) and Visible Infrared Imaging Radiometer Suite (VIIRS) Emissions (RAVE) (2023); https://doi.org/10.25921/9X2B-8T20.

55. K. Suselj, M. Kurowski, B. Samuels, B. H. Kahn, M. J. Chinita, J. Teixeira, Coupled weather-fire simulation of the 2025 Eaton Fire at the wildland–urban interface. ESS Open Archive [Preprint] (2025). https://doi.org/10.22541/essoar.176384759.95874166/v1.

56. C. Gerbig, J. C. Lin, S. C. Wofsy, B. C. Daube, A. E. Andrews, B. B. Stephens, P. S. Bakwin, C. A. Grainger, Toward constraining regional-scale fluxes of $CO_2$ with atmospheric observations over a continent: 2. analysis of COBRA data using a receptor-oriented framework. *J. Geophys. Res. Atmos.* **108**, 4757 (2003).

57. C. Gerbig, S. Körner, J. C. Lin, Vertical mixing in atmospheric tracer transport models: Error characterization and propagation. *Atmos. Chem. Phys.* **8**, 591–602 (2008).

58. Optimizing Smoke and Plume Rise Modeling Approaches at Local Scales; https://mdpi.com/2073-4433/9/5/166.

59. I. M. Ulbrich, M. R. Canagaratna, Q. Zhang, D. R. Worsnop, J. L. Jimenez, Interpretation of organic components from positive matrix factorization of aerosol mass spectrometric data. *Atmos. Chem. Phys.* **9**, 2891–2918 (2009).

60. Q. Zhang, J. L. Jimenez, M. R. Canagaratna, I. M. Ulbrich, N. L. Ng, D. R. Worsnop, Y. Sun, Understanding atmospheric organic aerosols via factor analysis of aerosol mass spectrometry: A review. *Anal. Bioanal. Chem.* **401**, 3045–3067 (2011).

61. A. V. Polissar, P. K. Hopke, R. L. Poirot, Atmospheric aerosol over Vermont: Chemical composition and sources. *Environ. Sci. Technol.* **35**, 4604–4621 (2001).

62. U.S. Environmental Protection Agency, EPA Positive Matrix Factorization (PMF) 5.0 Fundamentals and User Guide (2014); https://cfpub.epa.gov/si/si_public_record_report.Cfm?Lab=NERL&direntryid=308292, office of Research and Development, National Exposure Research Laboratory.

63. J. Camman, B. Chazeau, N. Marchand, A. Durand, G. Gille, L. Lanzi, J.-L. Jaffrezo, H. Wortham, G. Uzu, Oxidative potential apportionment of atmospheric $PM_1$: A new approach combining high-sensitive online analysers for chemical composition and offline OP measurement technique. *Atmos. Chem. Phys.* **24**, 3257–3278 (2024).
